# MCM10, a novel YAP1/TEAD4 target, drives gastric cancer progression by bridging DNA replication to stemness acquisition

**DOI:** 10.1186/s12943-026-02623-8

**Published:** 2026-02-27

**Authors:** Fuda Xie, Hoi Wing Leung, Yang Lyu, Peiyao Yu, Tiejun Feng, Bonan Chen, Jialin Wu, Jenson Tham, Canbin Fang, Alvin H.K. Cheung, Chit Chow, Jianhui Jiang, Jintao Hu, Fengbin Zhang, Chaowei Zhu, Keli Zhong, Meiheng Sun, Ge Zhang, Sifan Yu, Dazhi Xu, Shouyu Wang, Bing Huang, Kangmin Zhuang, Xiaobei Luo, Aimin Li, Qing Guo, Chanchan Gao, Bin Zhang, Yuan Ma, William KK Wu, Liwei An, Chi Chun Wong, Jun Yu, Ka Fai To, Wei Kang

**Affiliations:** 1 Department of Anatomical and Cellular Pathology, State Key Laboratory of Translational Oncology, Prince of Wales Hospital, The Chinese University of Hong Kong, Hong Kong, China; 2 State Key Laboratory of Digestive Disease, Institute of Digestive Disease, The Chinese University of Hong Kong, Hong Kong, China; 3 CUHK-Shenzhen Research Institute, The Chinese University of Hong Kong, Shenzhen, China; 4 Guangdong Provincial Key Laboratory of Malignant Tumor Epigenetics and Gene Regulation, Medical Research Center, Sun Yat-Sen Memorial Hospital, Sun Yat-Sen University, Guangzhou, China; 5 Department of Pathology, Shenzhen People’s Hospital, The Second Clinical Medical College, Jinan University, Shenzhen, Guangdong China; 6 Department of Gastroenterology, The Fourth Hospital of Hebei Medical University, Shijiazhuang, China; 7 Department of Gastrointestinal Surgery, The Second Clinical Medical College, The First Affiliated Hospital, Shenzhen People’s Hospital, Jinan University, Southern University of Science and Technology), Shenzhen, Guangdong China; 8 Law Sau Fai Institute for Advancing Translational Medicine in Bone and Joint Diseases (TMBJ), School of Chinese Medicine, Hong Kong Baptist University, Hong Kong, China; 9 Department of Gastric Surgery, Department of Oncology, Shanghai Medical College, Fudan University Shanghai Cancer Center, Fudan University, Shanghai, China; 10 Department of Hepatobiliary Surgery, Anhui Provincial Innovation Institute for Pharmaceutical Basic Research, Anhui Province Key Laboratory of Tumor Immune Microenvironment and Immunotherapy, The First Affiliated Hospital of Anhui Medical University, Innovative Institute of Tumor Immunity and Medicine (ITIM), Hefei, China; 11 Guangdong Provincial Key Laboratory of Gastroenterology, Department of Gastroenterology, Nanfang Hospital, Southern Medical University, Guangzhou, China; 12 Taizhou School of Clinical Medicine, The Affiliated Taizhou People’s Hospital of Nanjing Medical University, Nanjing Medical University, Nanjing, China; 13 Department of Oncology, Zhongda Hospital, Southeast University School of Medicine, Southeast University, Nanjing, China; 14 Department of Gastroenterology, Affiliated Hospital of Medical School, Nanjing Drum Tower Hospital, Nanjing University, Nanjing, China; 15 School of Chinese Medicine, The Chinese University of Hong Kong, Hong Kong, China; 16 Department of Anaesthesia and Intensive Care, The Chinese University of Hong Kong, Hong Kong, China; 17 Department of Stomatology, Department of Biochemistry and Molecular Biology, Shanghai Tenth People’s Hospital, Tongji University School of Medicine, Shanghai, China; 18 Department of Medicine and Therapeutics, The Chinese University of Hong Kong, Hong Kong, China; 19 Department of Anatomical and Cellular Pathology, The Chinese University of Hong Kong, Hong Kong, 999077 China

**Keywords:** Gastric cancer, MCM10, DNA replication, cancer cell stemness, TEAD4

## Abstract

**Objectives:**

Gastric cancer (GC) remains a major global health challenge, with chemotherapy resistance significantly hindering treatment efficacy. A significant proportion of chemotherapeutics impact DNA replication, yet the mechanisms by which tumors evade this lethality remain incompletely understood. Notably, minichromosome maintenance 10 replication initiation factor (MCM10) is pivotal in initiating DNA replication, holding promise in mediating acquired chemotherapy resistance. This work aims to elucidate the driving roles of MCM10 GC pathogenesis and chemotherapeutic resistance.

**Methods:**

The expression pattern of MCM10 and its clinical relevance in GC patients were investigated by adopting single-cell RNA-seq data and in-house GC tissue microarray. Functional roles were evaluated through bioinformatic analyses and experimental assays, including in vivo xenograft formation assay and patient-derived organoid (PDO) models. The transcriptional regulation of MCM10 by the YAP1-TEAD4 complex was examined via *Yap1*^*−/−*^;*Taz*^*−/−*^ transgenic mice models and functional rescue assays. Candidates for targeting MCM10 were predicted by virtual screening and further validated by cellular thermal shift assay (CETSA).

**Results:**

MCM10 was the most upregulated MCM family member in GC cell lines, and its elevated levels correlated with poor patient prognosis. Bioinformatic analysis linked MCM10 to DNA replication and DNA damage repair, a finding confirmed by functional assays showing that MCM10 depletion induced DNA damage accumulation and impaired DNA replication. MCM10 was further proven to promote GC cell malignancy and tumorigenesis by activating Wnt/β-catenin signaling in GC cell lines, clinical samples, and xenograft models. Critically, MCM10 conferred resistance to chemotherapeutic agents by enhancing cancer cell stemness acquisition and DNA damage response. Mechanistically, YAP1/TEAD4 was identified as the transcriptional activator of MCM10, as TEAD4 silencing downregulated MCM10. TEAD4 overexpression failed to rescue the tumor-suppressing effects in MCM10-depleted cells. Furthermore, Momordin Ic was identified as a promising MCM10-targeted inhibitor, which effectively attenuated GC cell malignancy and chemoresistance.

**Conclusion:**

MCM10 drives gastric tumorigenesis by enhancing DNA replication and maintaining cancer stemness, positioning it as a key mediator of YAP1-TEAD4 oncogenic signaling. These findings establish MCM10 as a promising therapeutic target to overcome chemotherapy resistance in GC.

**Graphical Abstract:**

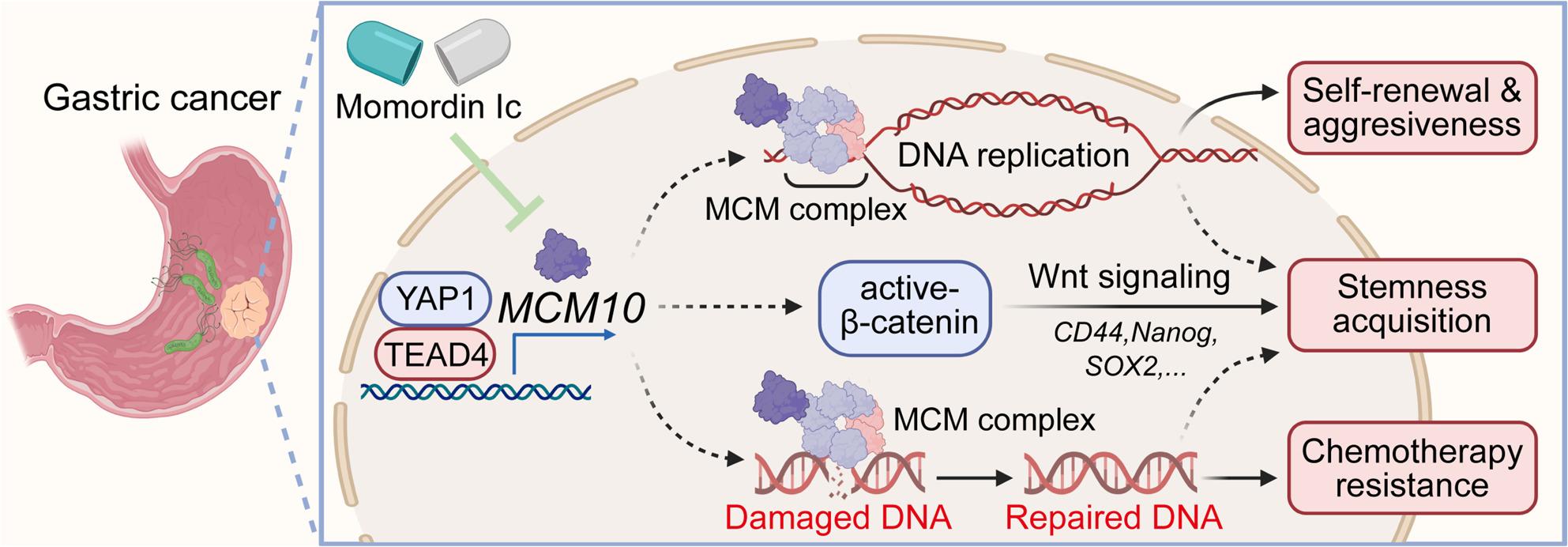

**Supplementary Information:**

The online version contains supplementary material available at 10.1186/s12943-026-02623-8.

## Introduction

Gastric cancer (GC) has long been recognized as a major health threat, impacting millions of lives worldwide [1]. Despite a marked decline in its mortality rate over the past decades, it remained the fifth most frequently diagnosed cancer and the fourth leading cause of cancer-related death globally, according to the Global Cancer Statistics 2022 [2]. Especially in certain Asian countries, GC ranks as the most frequent cancer and the leading cause of cancer death [2]. Recent studies highlighted the acquired resistance of chemotherapy as one of the primary causes of cancer-related deaths of GC patients [3, 4]. Thus, understanding the detailed mechanisms underlying chemoresistance during GC progression is crucial for developing effective treatment strategies.

Increasing studies suggested that the acquisition of chemoresistance in GC patients is largely regulated by hyperactivated DNA damage repair processes [5, 6]. First-line chemotherapeutic agents such as 5-fluorouracil (5-FU) exert their anticancer effects primarily by inhibiting DNA replication and repair, ultimately suppressing tumor cell proliferation [7]. However, hyperactivation of DNA damage response pathways can counteract these effects, leading to therapeutic resistance [6]. Cancer stem cells (CSCs) were demonstrated to further enhance the resistance of the GC microenvironment (TME) against chemotherapy [8]. The hyper-activated oncogenes responsible for CSC self-renewal and proliferation, such as CD44, OCT4, and CD133, would further compromise the intended effects of chemotherapy in reducing cancer cell populations [9, 10]. Mechanistically, the aberrant activation of Yes-associated protein 1 (YAP1) signaling contributes significantly to the transcriptional activation of CSC signatures [11, 12]. YAP1 activity is normally suppressed by the Mst1/2-SAV1-Lats1/2 cascade. During tumorigenesis, this pathway is frequently disrupted, resulting in YAP1 nuclear translocation to bind with TEA domain transcription factors (TEADs). The YAP1/TEADs transcription complex can bind to specific DNA elements and recruit chromatin modifiers to drive the expression of a pro-proliferative and pro-survival gene program [13, 14]. In GC, YAP1 was reported to increase cancer cell stemness via SOX9 during peritoneal metastasis [15, 16]. Inhibition of YAP1/TEADs can reduce the CSC subpopulation and impair the tumor sphere formation efficiency [17]. However, the molecular mechanisms of YAP1/TEADs in regulating cancer stemness remain unclear.

Accumulating evidence indicates that DNA replication initiation and licensing factors play crucial roles in cancer progression and the development of chemoresistance [18]. Several of these factors have been identified as significant prognostic markers across various cancer types [19, 20]. Minichromosome maintenance (MCM) family proteins were highlighted to play significant roles in various types of malignancies, including minichromosome maintenance 10 replication initiation factor (MCM10), an essential protein for the initiation of genome replication in metazoans [21]. Although the importance of MCM10 for viability varies in some model organisms like *Arabidopsis thaliana* [22], it is essential in mammalian systems, including human cells, for assembling and activating the replicative helicase. During DNA replication, the activation of the MCM2-7 helicase requires the binding of the helicase-activating proteins Cdc45 and GINS, which together form the replicative Cdc45-MCM-GINS (CMG) helicase complex. However, DNA unwinding cannot occur until MCM10 is associated with the complex [23, 24]. Consequently, MCM10 continues to move along with the replication fork after facilitating the initial DNA unwinding [25]. MCM10 is regulated by proteolysis and phosphorylation in a cell cycle-dependent manner, and it binds to chromatin exclusively during the S phase of the cell cycle [26]. Since MCM10 is structurally and functionally distinct from other MCM members, the exact mechanisms by which MCM10 influences cancer systems are unclear.

This study focuses on investigating the role of MCM10 in GC progression and elucidating its potential in regulating chemoresistance. We firstly identified MCM10 as the most upregulated MCM protein and found its expression to be closely correlated with poorer prognosis of GC patients, which aligns with the growing recognition of MCM10 as a critical oncoprotein across diverse malignancies [27]. Mechanistic analysis highlighted the critical role of the Wnt signaling pathway in MCM10-mediated CSC proliferation and acquisition of chemoresistance. Furthermore, this is the first study to demonstrate that the YAP1/TEAD4 co-transcription factor is a direct upstream regulator of MCM10. Importantly, we identified a small molecular candidate targeting MCM10-mediated CSC proliferation, which holds promise for blocking the acquisition of chemoresistance during GC treatment.

## Materials and methods

### GC cell lines, organoids, and primary sample cohort

Human gastric tumor cell lines (MKN1, MKN28, MKN45, MKN7, SNU16, SNU1, AGS, NCI-N87, KatoIII) and normal gastric epithelial cell line, GES-1 were purchased from American Type Culture Collection (ATCC). mRNA expression microarray was completed according to Two-Color Microarray-Based Gene Expression Analysis, Agilent Technologies, and the data was generated by GeneSpring GX Software. Details for the cell culture procedure were described in our previous study [28]. Briefly, cells were cultivated at 37 °C in a humidified environment, which contained 5% CO_2_. RPMI 1640 (GIBCO) was added with 10% fetal bovine serum (FBS, GIBCO). The culture of GC patient-derived organoid (PDO) models and gastric organoids derived from the stomach of Tff1-KO mice was cultured in accordance with previously instituted protocol [29] and as follows: GC tumor tissues were collected, rinsed, minced, and incubated at 37℃ for 1 h. The suspension was quenched using a cold culture medium, followed by filtration through a 70-micrometer strainer and then centrifugation at 400 g for 5 min. Cell pellets were resuspended with Matrigel (BD Biosciences) and seeded in the well to establish a 3D culture model. The Hong Kong GC tissue microarray cohort comprises 278 GC cases collected at Prince of Wales Hospital between 2002 and 2014. The use of human samples was approved by the Joint Chinese University of Hong Kong-New Territories East Cluster Clinical Research Ethics Committee, Hong Kong (CREC Ref. No.: 2022-060).

### In vitro functional assays

Cell transfections in this work were carried out using Lipofectamine 2000 Transfection Reagent (ThermoFisher Scientific). Depletion of MCM10 and TEAD4 was achieved via transient transfection with gene-specific small interfering RNAs (siRNAs) or stable transduction with lentiviral short hairpin RNAs (shRNAs, pLKO.1 backbone). Overexpression was performed by transfection with plasmid vectors carrying the target gene open reading frame. All siRNA, shRNA, and plasmid information is detailed in Supplementary Tables S1-S3. The information of primary and secondary antibodies used in the Western blot was recorded in Supplementary Table S4. Detailed procedures of functional assays, such as monolayer colony formation, cell invasion, and spheroid formation assays, were recorded in Supplementary Materials and Methods.

### Immunofluorescence (IF) assay

Cells adhered to the slides were initially fixed with 4% paraformaldehyde (PFA) and subsequently blocked with a phosphate-buffered saline (PBS) solution containing 0.1% Triton X-100 (Sigma-Aldrich) and 1% BSA. The slides were incubated overnight at 4 °C with primary antibody active β-catenin (1:100, Cell Signaling Technology, #8814), followed by incubation for 1 h at room temperature with the secondary antibody Alexa Fluor 488-Goat anti-Rabbit IgG (H + L) (1:500, A-11008, Thermo Fisher). For nuclear staining, the sections were treated with 1 µg/mL DAPI (1:10, FP1490, AKOYA BIOSCIENCES) for 15 min at room temperature. Imaging was performed using a Zeiss LSM 880 confocal microscope.

### Immunohistochemistry (IHC) staining

Immunohistochemistry was performed on tissue microarrays and cell-derived xenografts using a Ventana NexES automated Stainer (Ventana Corporation). Following de-waxing in xylene, all sections were subjected to microwave heating in EDTA antigen retrieval buffer. The protocol was detailed in our previous report [28]. Information of antibodies adopted in IHC staining was provided in Supplementary Table S5. For the primary human GC tissue microarray (Hong Kong cohort), the expression of MCM10 and TEAD4 was initially assessed and classified as “high” or “low” based on the percentage of positively stained tumor cells by two independent pathologists from Prince of Wales Hospital. The immunoreactive score for protein expression was quantified by assessing both the percentage of positively stained tumor cells and the staining intensity. These analyses were conducted using ImageJ software and the results have been recorded in Supplementary Table S6.

### RNA-sequencing (RNA-seq) analysis

Total RNA was extracted from MCM10- or TEAD4-depleted cells using the RNeasy kit (Qiagen), and its quality was assessed with a Tapestation (Agilent). Library preparation was performed using the Illumina Truseq RNA Kit (Illumina), followed by sequencing on the NovaSeq 6000 platform (Illumina) with single-end reads of 100 bp. Reads were quality-checked with FastQC (v0.12.0), and sequence trimming was conducted using Cutadapt (v4.2). The raw sequencing reads were aligned to the Homo sapiens genome assembly GRCh37 (hg19) from the NCBI database using HISAT2 (v2.1.0), and gene expression was quantified with FeatureCount (v1.6.4). Differentially expressed genes (DEGs) were identified using the R package “DESeq2” (v1.38.1), and enrichment analysis was conducted with “ClusterProfiler” (v4.6.2). Additionally, the processed bulk RNA-seq data generated in this study are provided in Supplementary Table S7.

### Public dataset-based bioinformatic analysis

Two public GC cohorts were adopted in this study: The Cancer Genome Atlas-stomach adenocarcinoma cohort (TCGA-STAD) [30] and the Asian Cancer Research Group (ACRG) cohort (GSE66229) [31]. For the functional enrichment analysis regarding the *MCM10* expression level, we first evaluated the whole genomic expression level alteration by comparing the 10% samples with the highest *MCM10* expression (*MCM10*^*+*^ samples) and 10% samples (*n* = 37) with the lowest *MCM10* expression (*MCM10*^*−*^ samples). DEGs were identified by R package “DESeq2”, and the enrichment analysis were performed using R package “ClusterProfiler”. Pearson’s correlation analysis was conducted based on data from TCGA, ACRG, Cancer Cell Line Encyclopedia (CCLE, https://sites.broadinstitute.org/ccle/) and calculated by R package “stats” (v4.1.3). The binding motifs of YAP1/TEAD4 on the corresponding promoter region of *MCM10* were predicted by the Eukaryotic Promoter Database (https://epd.epfl.ch//index.php) and JASPAR 2022 (https://jaspar.genereg.net). Drug resistance analysis was based on Genomics of Drug Sensitivity in Cancer (GDSC, https://www.cancerrxgene.org/) database [32] and a published RNA-seq dataset (https://www.ncbi.nlm.nih.gov/bioproject/PRJNA591481) [33].

### Single-cell RNA-seq (scRNA-seq) analysis

The scRNA-seq analysis was performed using the R package “Seurat” (v4.0.2) based on a public GC dataset (https://dna-discovery.stanford.edu/research/datasets/, “Gastric scRNAseq” dataset) [34]. Cells expressing fewer than 200 genes, having more than 20% mitochondrial genes, or exhibiting an outlier number of unique molecular identifiers were removed. Genes detected in fewer than 3 cells were also excluded. Following data normalization and cluster labeling, gene expression in individual cells was visualized with “FeaturePlot”, “DimPlot”, “DotPlot”, and “VlnPlot”. The gene markers for cluster labeling and corresponding references are listed in Supplementary Table S8. Gene Set Variation Analysis (GSVA) was conducted using the R package “GSVA” (v1.46.0), while the relative differentiation state of cells was predicted through CytoTRACE (v0.3.3). Additionally, single-cell pseudotime analysis was carried out using Monocle2 within the “monocle” R package (v2.27.0). After filtering and dimension reduction according to the recommended parameters, cells were ordered and visualized using the “plot_cell_trajectory” and “plot_pseudotime_heatmap” functions. An additional GC scRNA-seq dataset [35] was analyzed using the same processing pipeline to validate the key findings generated in scRNA-seq analysis.

### Structure-based virtual screening

The 3D structure files of MCM10 were retrieved from Protein Data Bank (PDB, https://www.rcsb.org/) under the accession number “3H15”. The DNA-binding domain was determined by referring to former research [36]. The candidate compound library was composed of 2725 small molecules originating from traditional Chinese medicine with bioactivity. Molecular docking was performed by Autodock 4.2.6, and the Lamarckian genetic algorithm was applied for the docking procedure. The detailed parameters are listed in Supplementary Table S9. Information of the compound library and their corresponding binding affinities with MCM10 is provided in Supplementary Table S10.

### In vivo studies

The *Yap1* ^*floxed/+*^;*Taz* ^*floxed/+*^ murine line was obtained by crossing *Yap1* ^*floxed/+*^ mice (a kind gift from Professor Duojia Pan, UT Southwestern Medical Center) and *Taz* ^*floxed/+*^ mice (Biocytogen, Beijing, China). Genomic DNA extracted from tail biopsies was used to evaluate offspring genotype. *Ubc-Cre/ERT2* mice were purchased from the Jackson Laboratory. *Yap1*^*−/−*^;*Taz*^*−/−*^ mice were generated by cross-breeding *Yap1* ^*floxed/floxed*^; *Taz* ^*floxed/floxed*^ with the tamoxifen-inducible *Ubc-Cre/ERT2* mice. Tamoxifen induction at a dosage of 100 mg/kg was administered intraperitoneally three times per week for four weeks [37]. For subcutaneous xenograft formation assays, AGS cells (10^6^ cells/mouse) were treated with shCtrl or shMCM10 before being injected subcutaneously into 4-week-old NOD scid gamma (NSG) mice (*n* = 6/group). Tumor size was measured every two days using a digital caliper. The mice were sacrificed 16 days post-injection, and the xenografts were harvested, weighed, and processed for further IHC staining. To evaluate the synergistic effect of MCM10 deletion or Momordin Ic (MIc) with 5-fluorouracil (5-FU), four groups of NSG mice were subcutaneously inoculated with AGS cells (10^5^ cells/mouse) and treated with vehicle (PBS), shMCM10/MIc (15 mg/kg), 5-FU (10 mg/kg every 2 days, i.p.), or shMCM10/MIc + 5-FU. After 28 days of treatment, the mice were sacrificed, and the xenografts were harvested and evaluated. All mouse experiments were approved by the Animal Ethics Experimentation Committee (AEEC) at CUHK.

### Statistical analysis

Two tailed Student’s *t*-test was adopted to demonstrate the significance between assay groups and control in gene expression level comparison and the functional assays. Pearson’s correlation was used to conduct correlation analysis. Statistical analyses were performed by GraphPad Prism 8.0 (GraphPad). The displayed results showed the means and the SDs, and those with *P* values less than 0.05 were considered statistically significant (*, *P* < 0.05; **, *P* < 0.01; ***, *P* < 0.001). The Kaplan-Meier method was employed for the calculation of the survival rate, and the equivalence of the survival curve was checked by log-rank statistics.

## Results

### MCM10 is the most upregulated MCM family protein and correlates with poor survival of GC patients

**Fig. 1:**
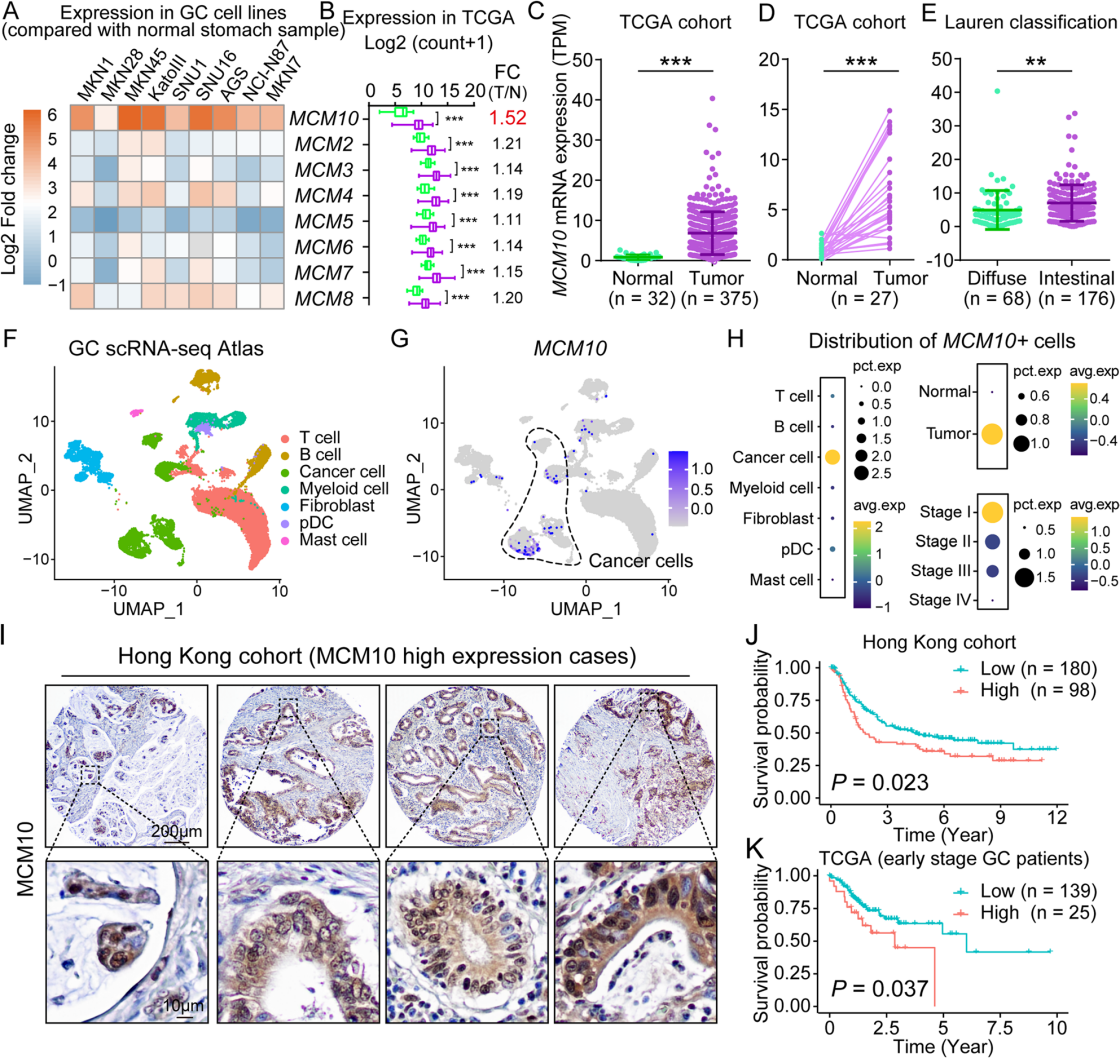
MCM10 is hyperactivated in GC tissues and predicts poorer clinical outcomes. **A** *MCM10* was the most upregulated protein in the MCM family across GC cell lines. **B** *MCM10* exhibited the highest variability among all MCM family members in the TCGA cohort. **C**-**D** *MCM10* mRNA levels were significantly higher in tumor tissues than in normal tissues. **E** Intestinal-type GC patients showed stronger *MCM10* expression compared to those with diffuse-type disease. **F**-**G** UMAP visualization of all cell types in the GC TME and cells with evaluated *MCM10* expression levels. **H** *MCM10 +* cells were enriched in the cancer cell subtype within the scRNA-seq atlas, particularly in tumor tissues during early stages. **I** Representative IHC staining images of MCM10 in GC samples from the Hong Kong cohort. **J**-**K** High MCM10 expression predicted worse prognosis in both the Hong Kong cohort and early stage GC patients from TCGA. (**, *P* < 0.01; ***, *P* < 0.001)

Compared with normal stomach samples, all GC cell lines demonstrated upregulated *MCM10* mRNA expression, and *MCM10* was found to be the most significantly upregulated member among the MCM family (Fig. 1A). Congruently, expression analysis based on the TCGA-STAD dataset also revealed *MCM10* as the most significantly upregulated MCM family protein (Fig. 1B). Consistently, *MCM10* mRNA levels were markedly higher in GC tissues compared to both paired and unpaired normal gastric tissues (Fig. 1C-D). Notably, the elevated expression of *MCM10* was observed in 9% (37 out of 407) cases within the TCGA cohort, while *MCM10* DNA amplification was identified in only 3 cases (Supplementary Fig. S1). Furthermore, higher expression levels of *MCM10* were observed in intestinal-type GC samples (Fig. 1E). In the GC scRNA-seq atlas, *MCM10* overexpression was enriched in cancer cells (Fig. 1F-G, Supplementary Fig. S2A-B). By integrating clinical information, we identified that *MCM10*-positive (*MCM10+*) cells were predominantly tumor cells, and largely enriched in stage I patients (Fig. 1H). Consistent with these findings, IHC staining of primary GC samples demonstrated that upregulated MCM10 protein was predominantly enriched in the nuclei of cancer cells, with variable cytoplasmic staining observed in some samples. (Fig. 1I). Notably, clinical significance analysis revealed that high MCM10 expression was associated with poor prognosis in GC patients (Fig. 1J), particularly those diagnosed at early stages of the disease (Fig. 1K). In contrast, MCM10 expression lost its prognostic significance in late stage GC patients (Supplementary Fig. S3), suggesting its role as a marker of intrinsic tumor aggressiveness is more powerful in early stage disease, before the clinical course is complicated advanced, metastatic illness.

### MCM10 plays a significant role in GC cell malignancy and tumorigenesis

**Fig. 2:**
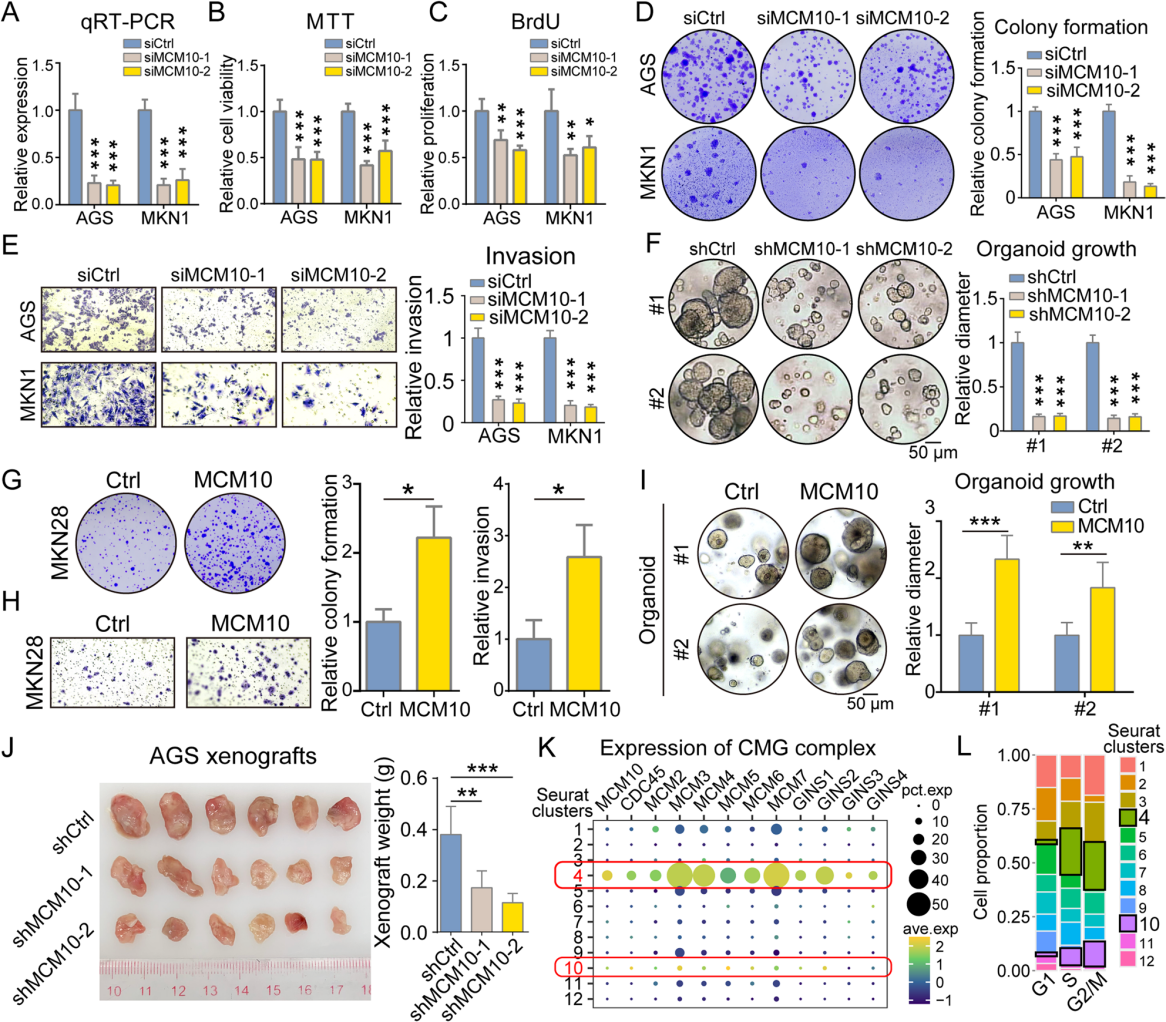
MCM10 promotes malignant phenotypes in GC cells, organoids, and xenograft models. **A** siRNA-mediated knockdown of MCM10 effectively decreased its mRNA expression in AGS and MKN1 cells (*n* = 5). **B-C** MCM10 depletion downregulated the cell viability and DNA synthesis in GC cell lines (*n* = 5). **D-E **MCM10-depletion significantly impaired colony formation and invasive capacity (*n* = 3). **F** The growth of GC organoids was restrained by the knockdown of MCM10 (*n* = 5). **G-I** Overexpression of MCM10 enhanced the colony formation and invasion ability of MKN28 (*n* = 3), as well as the growth of organoids (*n* = 5). **J** MCM10 overexpression accelerated in vivo xenograft formation (*n*  = 6). **K-L** Expression levels of components in the CMG complex showed coordinated upregulation within the same Seurat clusters. Two specific cell clusters (cluster 4 and 10) represent subpopulations of cancer cells showcasing high expression of all CMG components and predominant enrichment in the S and G2/M phases of the cell cycle (*, *P* < 0.05; **, *P* < 0.01; ***, *P* < 0.001)

To examine whether MCM10 functionally drives cancer cell malignancy and tumor growth, MCM10-knockdown experiments were performed. The qRT-PCR analysis demonstrated successful establishment of knockdown clones derived from AGS and MKN1 GC cells (Fig. 2A). The knockdown of MCM10 significantly reduced cell proliferation in AGS and MKN1, as determined by MTT assay (Fig. 2B), and inhibited DNA synthesis in proliferating cells, as shown by the BrdU assay (Fig. 2C). Consistently, MCM10 depletion significantly and dramatically decreased colony formation and invasion of GC cells (Fig. 2D-E). Furthermore, MCM10 depletion significantly restrained the growth of GC organoids derived from two clinical samples (Fig. 2F). Similarly, in the overexpression assays, upregulated MCM10 level significantly enhanced colony formation and invasion of MKN28 cells (Fig. 2G-H). In addition, the growth of GC organoids and cell-derived xenografts (CDXs) was remarkably accelerated by overexpressing MCM10 (Fig. 2I-J), strongly suggesting an essential role of MCM10 in regulating tumor growth. The expression patterns of CMG complex members were further investigated, and the results indicated a common enrichment in *MCM10 +* cells, with coordinated upregulation observed within the same Seurat clusters (Fig. 2K). Furthermore, *MCM10 +* cells exhibited significant enrichment in S and G2/M phases of the cell cycle, underscoring a pivotal role for MCM10 in driving cell cycle progression during GC progression (Fig. 2L).

### MCM10 regulates cell cycle progression in GC

**Fig. 3:**
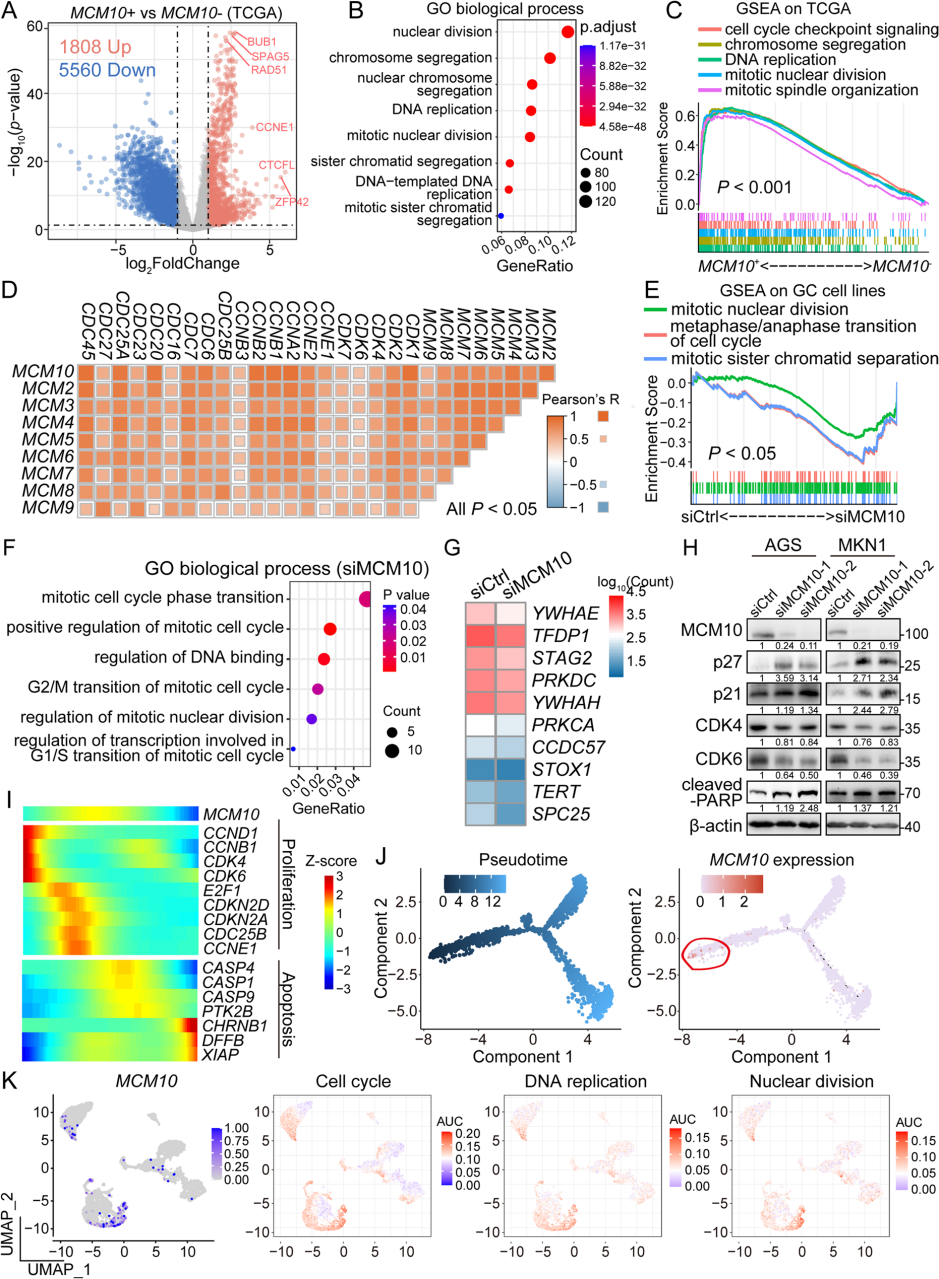
MCM10 played a crucial role in the regulation of cell cycle progression. **A**-**B** Various genes and signaling pathways involved in the cell cycle process were enriched in *MCM10 +* GC samples. **C** GSEA indicated a significant enrichment of cell cycle-related pathways in *MCM10 +* GC cases. **D** Pearson correlation analysis revealed coordinated expression among MCMs, CDKs, cyclins, and other cell cycle regulators. **E**-**F** Knockdown of MCM10 suppressed the activity of key cell cycle pathways. **G** MCM10-depletion downregulated the expression of multiple cell cycle markers. **H** Western Blot results demonstrated cell cycle arrest features after MCM10-depletion. **I**-**J** Pseudotime trajectory analysis revealed that *MCM10* upregulation occurred in the early stages of cancer cell development, concomitant with the expression of proliferation markers. **K** scRNA-seq data further confirmed a strong association between *MCM10* expression and signatures of cell cycle, DNA replication, and nuclear division

In order to uncover the oncogenic mechanisms of MCM10 in GC progression, analysis on TCGA-STAD dataset was first conducted. The DEG analysis highlighted 1,808 upregulated genes and 5,560 downregulated genes in *MCM10 +* samples, with large expression differences and high statistical significance. The most significant upregulated genes were identified as cell cycle-related genes, including *BUB1*, *SPAG5*, and *RAD51* (Fig. 3A). The enrichment analysis based on the upregulated genes revealed a close correlation with biological processes involved in cell cycling, such as nuclear division, chromosome segregation, and DNA replication (Fig. 3B). The Gene Set Enrichment Analysis (GSEA) results also demonstrated upregulated activity of pathways like cell cycle checkpoint signaling and mitotic spindle organization in *MCM10 +* samples, indicating a hyper-activated cell cycling (Fig. 3C). A comprehensive correlation analysis revealed close associations between MCM family proteins and cell cycling regulators (Fig. 3D). Consistently, RNA-seq analysis on the MCM10-delepted GC cell lines revealed downregulated activities of cell cycle-related biological processes (Fig. 3E-F) and gene signatures (Fig. 3G). The regulatory role of MCM10 in the cell cycle was further confirmed by Western blot analysis. The results demonstrated that MCM10 knockdown downregulated CDK4/6 and upregulated p21/p27, indicating a G1 cell cycle arrest in MCM10-depleted cell lines (Fig. 3H). Upregulated cleaved-PARP in MCM10-depleted cell lines also suggests an increased induction of apoptosis. In single-cell resolution, *MCM10* upregulation was chronologically correlated with proliferation markers, while apoptosis markers were upregulated in the late stage of the cell cycle. This dynamic pattern further confirms the functional role of *MCM10*, demonstrating its specific activity is confined to the proliferative cell state and is absent once cells commit to an apoptotic fate. (Fig. 3I). Besides, *MCM10 +* cells were enriched in the early stage of the pseudotime trajectory, suggesting a role of MCM10 in the initiation of cell proliferation (Fig. 3J, Supplementary Fig. S2C-D). Further GSVA results also revealed that *MCM10 +* cells exhibited relatively higher activity in cell cycle-related biological processes (Fig. 3K, Supplementary Fig. S2E).

### MCM10 participates aggressively in the maintenance of GC cellular stemness

**Fig. 4:**
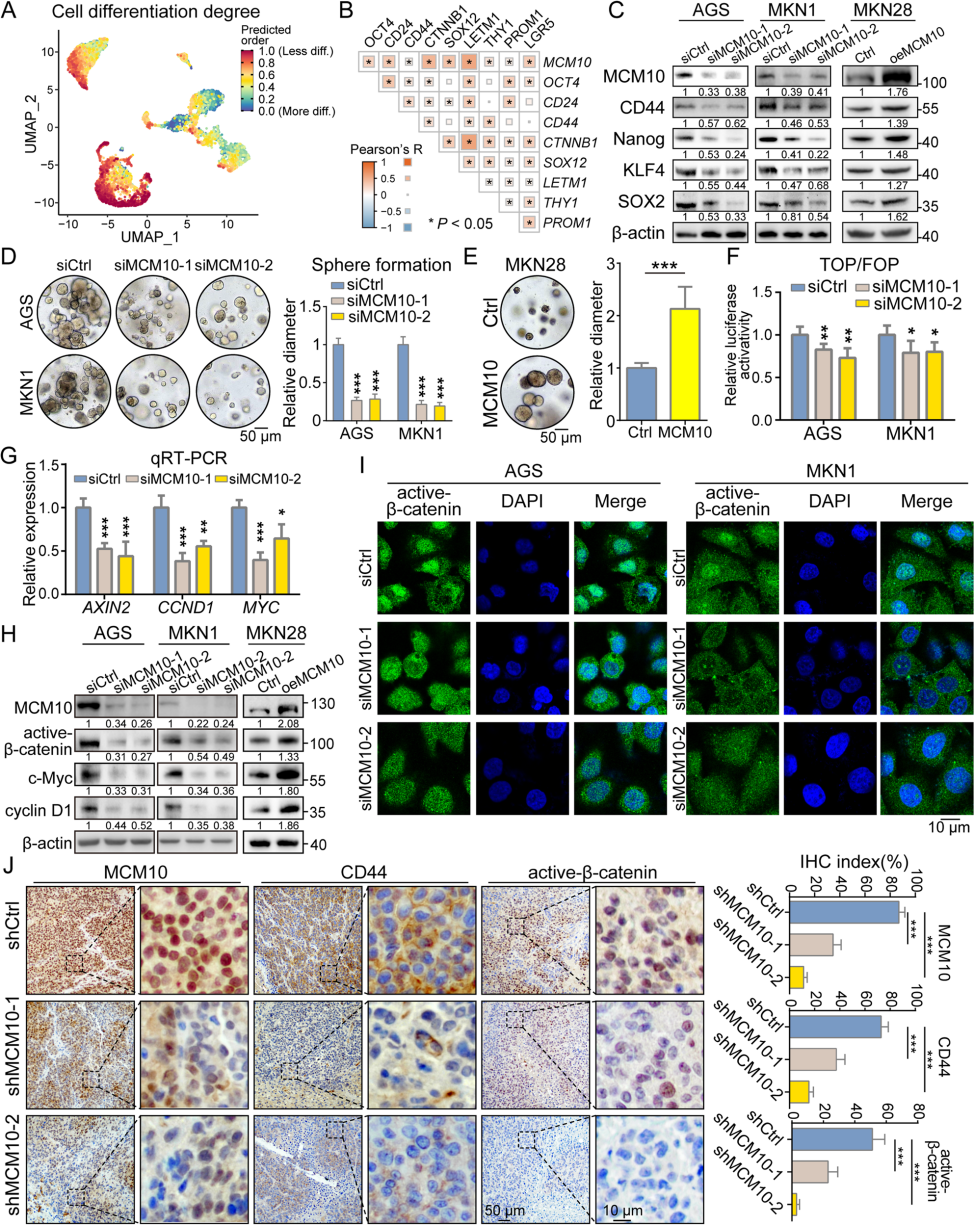
MCM10 promotes cancer cell stemness through Wnt/β-catenin signaling activation. **A** scRNA-seq analysis revealed that *MCM10 +* cells exhibit less differentiated state. **B** *MCM10* expression was positively correlated with multiple stemness markers in TCGA cohort. **C** Knockdown of MCM10 downregulated the expression of classic stemness markers, while overexpression of MCM10 upregulated them. **D**-**E** MCM10 depletion impaired the spheroid formation ability of GC cell lines, whereas overexpression enhanced this stemness-associated trait (*n* = 3). **F** Downregulated transcription activity of β-catenin was detected in MCM10-depleted CG cells (*n* = 3). **G**-**H** Downstream targets of β-catenin revealed concordant downregulation in both mRNA and protein levels upon MCM10 knockdown, while MCM10 overexpression consistently increased their levels. **I** Knockdown of MCM10 restrained the nuclear localization of β-catenin (*n* = 3). **J** MCM10-depletion reduced CD44 and active β-catenin expression in mice xenografts (*n* = 3). (*, *P* < 0.05; **, *P* < 0.01; ***, *P* < 0.001)

A scRNA-seq-based cell differentiation stage analysis was conducted in the cancer cell subcluster. Stemness scoring analysis based on the CytoTrace algorithm showed that *MCM10 +* cells possessed a less differentiated state, suggesting their association with enhanced stemness properties (Fig. 4A, Supplementary Fig. S2F). Meanwhile, the TCGA-based expression correlation analysis demonstrated close correlation between *MCM10* expression and multiple cancer stem cell markers, such as *CD24*, *CD44*, and *PROM1* (encoding CD133) (Fig. 4B). Western blot analysis revealed that MCM10 depletion downregulated the expression of cancer cell stemness markers, including CD44, Nanog, KLF4, and SOX2, while the overexpression of MCM10 upregulated them (Fig. 4C). Furthermore, the self-renewal capacity, indicated by spheroid formation, was largely impaired by MCM10 knockdown, and enhanced by plasmid-mediated MCM10 overexpression (Fig. 4D-E). Flow cytometry analysis also showed that MCM10 knockdown decreased the percentages of cells indicating EpCAM and CD44 (Supplementary Fig. S4). As for the molecular mechanism, the Wnt/β-catenin signaling was long notoriously known for its promoting functions in cancer stem cell population maintenance, and was examined in this study. A TOP/FOP reporter assay revealed a decreased transcription activity of β-catenin in MCM10-depleted AGS and MKN1 cells (Fig. 4F). Consistent with the results for the transactivation activity of β-catenin, qRT-PCR analysis demonstrated that MCM10 knockdown downregulated the mRNA expression level of classic transcriptional downstream genes of β-catenin, including *AXIN2*, *CCND1* and *MYC* (Fig. 4G). This effect was also confirmed at the protein level, where knockdown of MCM10 significantly decreased the expression level of active β-catenin as well as the downstream targets c-Myc and cyclin D1, while MCM10 overexpression upregulated the protein expression level of the same markers (Fig. 4H). Remarkably, IF assay demonstrated a notable reduction in the proportion of nuclear accumulation of active β-catenin after knockdown of MCM10, strongly supporting the regulatory function of MCM10 in the Wnt/β-catenin signaling (Fig. 4I). IHC staining results further revealed reduced expression of the cancer stem cell surface marker CD44, along with diminished nuclear localization of active β-catenin in MCM10-depleted CDXs (Fig. 4J). To investigate the mechanisms by which MCM10 directly regulates β-catenin activity, IP-MS screening was conducted, and 27 proteins were identified as potential binding partners of MCM10 (Supplementary Fig. S5A). The detailed information on these binding partners was recorded in Supplementary Table S11. Among them, HSPA8 was highlighted as the most promising candidate for its close correlation with β-catenin nuclear activity and structural feasibility of binding with MCM10 (Supplementary Fig. S5B).

### Knockdown of MCM10 enhanced chemotherapeutic drug sensitivity via DNA damage induction

**Fig. 5:**
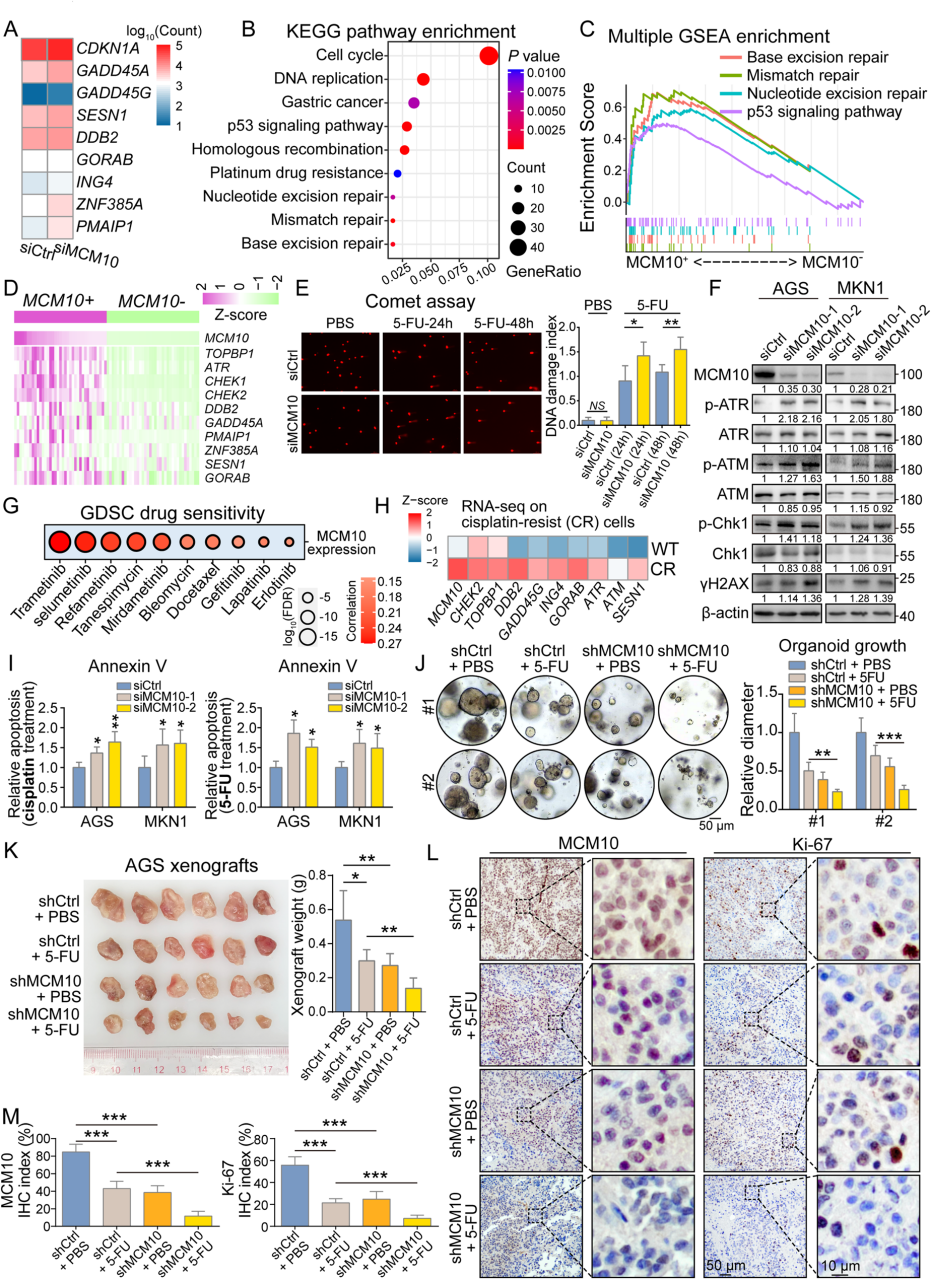
MCM10 mediates the DNA repair process and promotes resistance to chemotherapy. **A**-**B** MCM10 depletion activated DNA damage repair markers and signaling pathways. **C**-**D** *MCM10 +* samples exhibited enhanced activity of DNA repair gene signatures and associated biological processes. **E** Representative images and quantification of EdU+ cells in control and MCM10-knockdown GC cells (*n* = 3). **F** MCM10 knockdown induced activation of the DNA repair response. **G** *MCM10* expression was associated with predicted resistance to multiple chemotherapeutic agents. **H** Cisplatin-resistant cells showed concurrent upregulation of *MCM10* and DNA repair markers. **I** Knockdown of MCM10 sensitized GC cell lines to cisplatin and 5-FU treatment (*n* = 3). **J** MCM10-depletion showed a synergetic effect with 5-FU delivery in suppressing organoid growth (*n* = 5). **K** MCM10-deleted xenografts exhibited reduced tumor growth and increased sensitivity to 5-FU (*n* = 6). **L**-**M** Representative IHC images of xenograft sections stained by MCM10 and cancer cell proliferation marker Ki-67 (*n* = 3). (*NS*, not significant; *, *P* < 0.05; **, *P* < 0.01; ***, *P* < 0.001)

RNA-seq analysis revealed that MCM10 knockdown could activate the expression of various regulators involved in DNA damage repair (Fig. 5A). The KEGG pathway enrichment analysis based on the DEGs in siMCM10 group also indicated a significant alteration in cell cycle- and DNA damage repair-related pathways (Fig. 5B). The GSEA results indicated a significant enrichment of *MCM10 +* samples in multiple DNA repair-related pathways and the p53 signaling pathway (Fig. 5C). A closer look at the gene expression patterns in the TCGA dataset revealed that multiple DNA damage repair regulators were upregulated in *MCM10 +* samples (Fig. 5D). To directly test whether MCM10 depletion leads to the accumulation of DNA damage, as suggested by our transcriptomic data, we performed the comet assay. The results demonstrated MCM10 knockdown significantly reduced drug-induced DNA damage in AGS and MKN1 cells, providing direct evidence that MCM10 is essential for genomic integrity and confirming its functional role in the DNA damage response in GC (Fig. 5E). Western blot analysis also revealed that MCM10 depletion activated DNA damage repair pathways, including the ATM/ATR signaling cascade (Fig. 5F).

The GDSC dataset results indicated that *MCM10* expression is closely correlated with the drug resistance of multiple chemotherapy drugs, such as trametinib, selumetinib, and gefitinib (Fig. 5G). RNA-seq analysis of cisplatin-resistant AGS cells revealed that the expression level of *MCM10* and ATM/ATR signaling components was elevated in cells with acquired cisplatin resistance (Fig. 5H). To further confirm the role of MCM10 in chemo-sensitization, cytometry-based Annexin V assay was employed to quantify the relative resistance of GC cell lines to first-line chemotherapies. As the results suggested, suppression of MCM10 led to sensitization of the AGS and MKN1 to cisplatin/5-FU treatment (Fig. 5I and Supplementary Fig. S6). In the primary samples, MCM10-depletion significantly suppressed the growth of GC organoids and enhanced their sensitivity to 5-FU treatment (Fig. 5J). Consistent with the findings from the organoid assay, MCM10 knockdown remarkably improved the anti-tumor efficacy of 5-FU, as evidenced by substantially restricted xenograft formation in mice receiving combination therapy (Fig. 5K). IHC staining results indicated reduced expression of the cell proliferation marker Ki-67 following either 5-FU treatment or MCM10 depletion alone. Notably, the combination of 5-FU and MCM10-depletion showed superior efficacy in inhibiting tumor cell proliferation (Fig. 5L-M). We further conducted Annexin V assays to directly test whether elevated MCM10 is a driver of chemoresistance. The results showed that MCM10 overexpressing MKN28 cells exhibited a significant reduction in drug-induced apoptosis (Supplementary Fig. S7).

### TEAD4 is a novel direct upstream regulator of MCM10 in GC

**Fig. 6:**
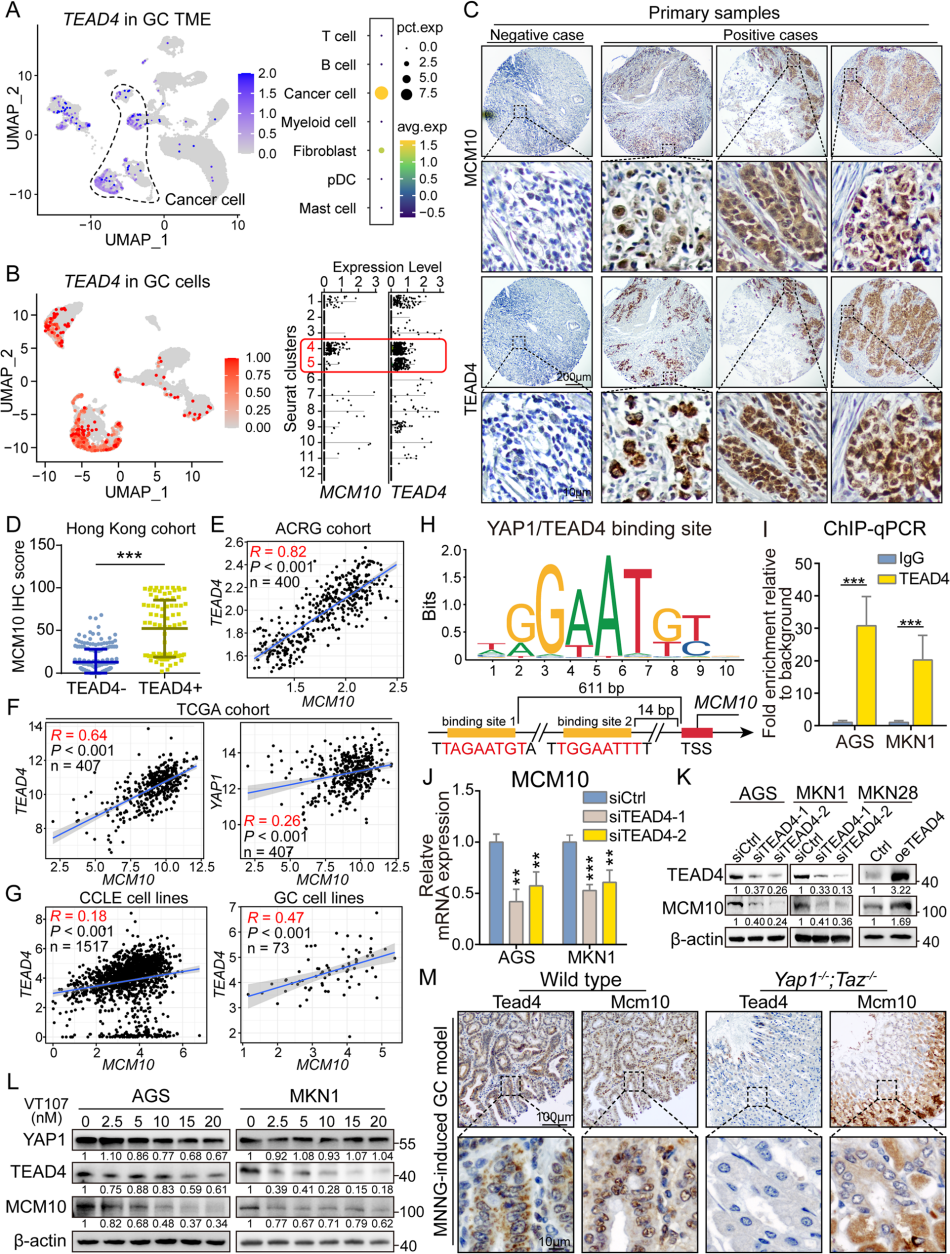
TEAD4 is identified as a transcriptomic regulator responsible for MCM10 activation. **A**-**B** scRNA-seq analysis revealed co-enrichment of *TEAD4 +* and *MCM10 +* cells within the same cancer cell clusters. **C** Representative IHC staining showing TEAD4 and MCM10 dual-negative and dual-positive GC samples. **D** MCM10 expression demonstrated significant upregulation in TEAD4 + samples. **E**-**F** The mRNA level of *MCM10* was positively correlated with *TEAD4* expression in both TCGA and ACRG cohorts. **G** Expression levels of *MCM10* and *TEAD4* showed consistent positive correlation across pan-cancer and GC-specific cell lines. **H**-**I** Putative YAP1/TEAD4 binding motifs were identified within the *MCM10* promoter region and subsequently validated by ChIP-qPCR (*n* = 3). **J**-**K** Depletion of TEAD4 led to downregulation of MCM10 expression, while its overexpression enhanced MCM10 levels. **L** Pharmacological inhibition of TEAD4 downregulated MCM10 expression. (M) Mcm10 expression was reduced in the gastric epithelial area in *Yap1*^*−/−*^;*Taz*^*−/−*^ transgenic mice. (**, *P* < 0.01; ***, *P* < 0.001)

Analysis on the GC scRNA-seq atlas revealed that *TEAD4*, a key transcription factor of the Hippo signaling pathway known to drive cell proliferation and stemness, was specifically upregulated in cancer cells of gastric tumors (Fig. 6A). Further analysis focusing on the cancer cell subclusters highlighted that the *MCM10 +* and *TEAD4 +* cells were enriched in the same clusters (Fig. 6B, Supplementary Fig. S2G-H). By IHC staining on our in-house GC tissue microarray, we identified a close correlation between the overexpression of MCM10 and TEAD4 in primary GC samples, and the co-upregulation of MCM10 and TEAD4 in the same cells was universally observed (Fig. 6C). Statistically, MCM10 expression was significantly upregulated in TEAD4 + samples (Fig. 6D). The correlation was further validated in multiple publicly accessible databases, including ACRG (Fig. 6E), TCGA (Fig. 6F), and CCLE databases (Fig. 6G). Taken together, our results consistently demonstrated a strong positive association between *MCM10* and *TEAD4* mRNA expression levels. Analysis based on the Eukaryotic Promoter Database also revealed the presence of two putative YAP1/TEAD4 binding sites in the promoter region of the *MCM10* gene at 611 bp and 14 bp ahead of the TSS (Fig. 6H). The direct binding between TEAD4 and *MCM10* promoter was further confirmed by Chromatin immunoprecipitation (ChIP)-qPCR (Fig. 6I), thereby establishing MCM10 as a direct transcriptional target of the TEAD4 oncogene and providing a mechanistic explanation for its upregulation in GC. Meanwhile, the ChIP-qPCR analysis also confirmed significant enrichment of YAP1 at the same putative TEAD4-binding site in the *MCM10* promoter region (Supplementary Fig. S8). Furthermore, qRT-PCR and Western blot analysis showed that both MCM10 mRNA (Fig. 6J) and protein (Fig. 6K) levels were consistently decreased in AGS and MKN1 cells following TEAD4 knockdown, while TEAD4 overexpression increased them. Additional qRT-PCR analysis confirmed that depletion of YAP1 led to a significant downregulation of *MCM10* mRNA levels (Supplementary Fig. S9). Treatment of VT107, a commercialized TEAD4-specific inhibitor, downregulated the expression level of TEAD4 and MCM10 in a dose-dependent manner, while YAP1 level remained unchanged (Fig. 6L). In murine models, TEAD4 depletion significantly restrained the organoid sizes (Supplementary Fig. S10A) and the expression of Mcm10 in mouse gastric organoids (Supplementary Fig. S10B), while overexpression of TEAD4 promoted organoid growth (Supplementary Fig. S10C). To provide a definitive assessment of the correlation between Tead4 transcriptional activation and Mcm10 expression while avoiding the potential compensating function of Taz in Yap1 knockout mice, *Yap1*^*−/−*^;*Taz*^*−/−*^ transgenic mice were adopted and treated with Methylnitronitrosoguanidine (MNNG) oral delivery for 6 months to generate gastric tumors. The nuclear expression of Mcm10 was significantly reduced in gastric epithelial areas from *Yap1/Taz*-deficient mice (Fig. 6M, Supplementary Fig. S11). Given the absence of significant tumor formation upon *Yap1/Taz* knockout, the tissue morphology was predominantly characterized by normal epithelium [37]. These data provided direct in vivo genetic evidence that the transcriptional activation of MCM10 is functionally dependent on the YAP1-TEAD4 signaling during gastric tumorigenesis.

### MCM10 is an indispensable functional mediator of the YAP1/TEAD4 transcription complex

**Fig. 7:**
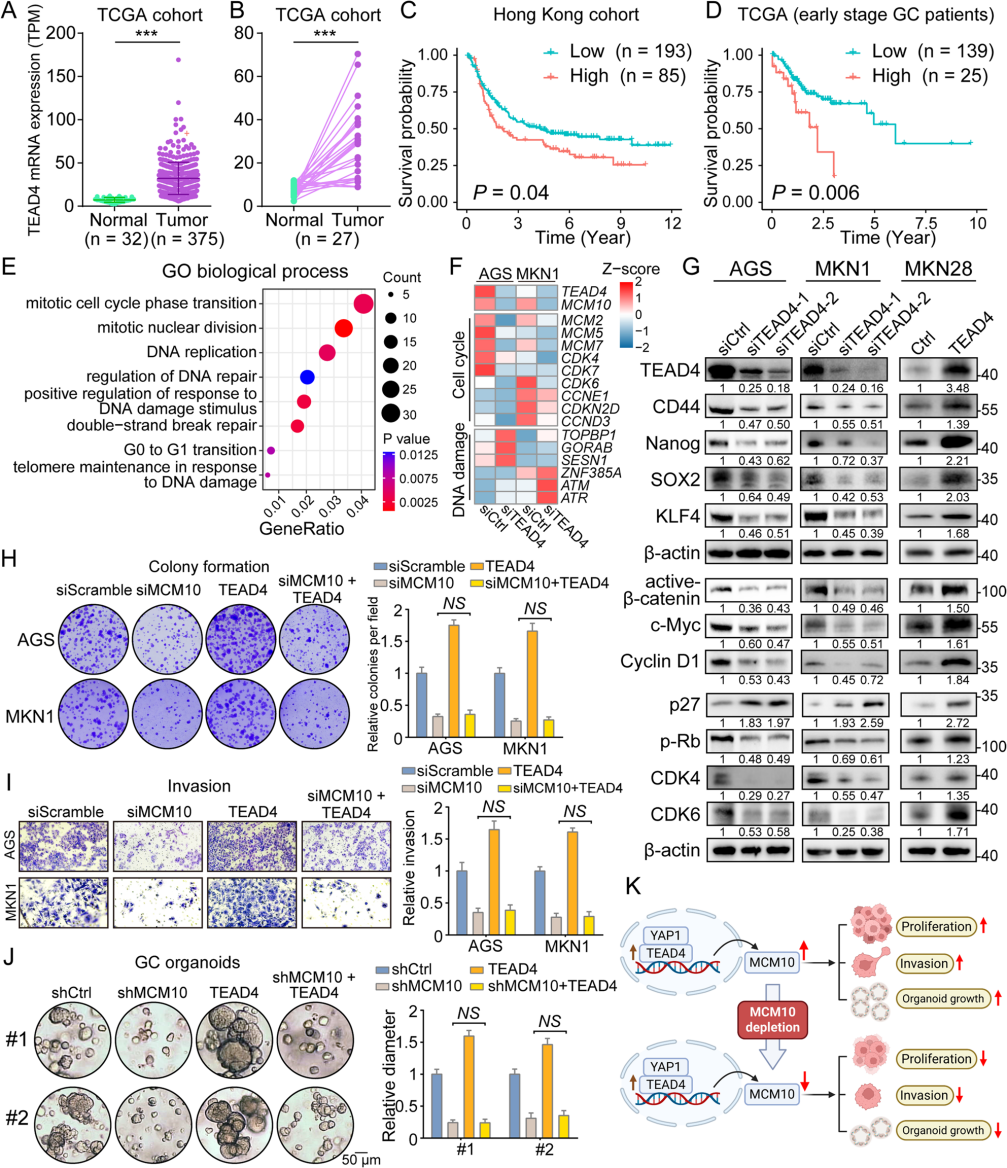
TEAD4 exerts oncogenic functions primarily through MCM10, and its high expression predicts poor prognosis. **A**-**B** *TEAD4* expression was significantly upregulated in tumor tissues compared to adjacent normal tissues. **C**-**D** High expression level of TEAD4 predicted poorer overall survival in both internal and public GC cohorts. **E**-**F** TEAD4 knockdown suppressed cell cycle progression and activated DNA damage response. **G** Expression changes in stemness markers, Wnt/β-catenin signaling components, and cell cycle regulators upon TEAD4 depletion or overexpression. **H**-**I** Overexpression of TEAD4 failed to rescue the impaired colony formation and invasion induced by MCM10 knockdown (*n* = 3). **J** While TEAD4 overexpression promoted organoid growth, concomitant MCM10 depletion abrogated this effect (*n* = 5). **K** Diagram scheme showcasing the YAP1/TEAD4-MCM10 axis in regulating tumor cell malignancy. (*NS*, not significant; ***, *P* < 0.001)

The expression of TEAD4 was largely upregulated in tumor samples compared to both paired and unpaired normal tissues (Fig. 7A-B). High TEAD4 mRNA level was correlated to poor prognosis in early stage GC patients (Fig. 7C). Similarly, evaluated protein expression of TEAD4 was also revealed as a poor prognosis indicator in the Hong Kong cohort (Fig. 7D). Functionally, knockdown of TEAD4 downregulated the expression of cell cycle regulators and initiated the activation of DNA repair process (Fig. 7E-F). Western blot analysis also validated that TEAD4 regulates stemness markers (CD44, Nanog, SOX2, KLF4), Wnt signaling signatures (active β-catenin, c-Myc, cyclin D1), and cell cycle progression (p27, pRb, CDK4/6) in GC cell lines (Fig. 7G). Rescue assays were subsequently conducted to confirm that MCM10 is essential for the TEAD4-mediated oncogenic function. As revealed by the colony formation and invasion assays, overexpression of TEAD4 led to exaggerated oncogenic phenotypes of GC cell lines AGS and MKN1, while MCM10 deletion inhibited these malignant features. Critically, this oncogenic function was dependent on MCM10, as the upregulation of TEAD4 was unable to promote cell proliferation and invasion when MCM10 was maintained at a low level (Fig. 7H-I). Similarly, the oncogenic function of TEAD4-overexpression on organoid growth and cell cycle progression was also blocked by the knockdown of MCM10 (Fig. 7J-K, Supplementary Fig. S12).

### Momordin Ic (MIc) is identified as a potent MCM10 inhibitor

**Fig. 8:**
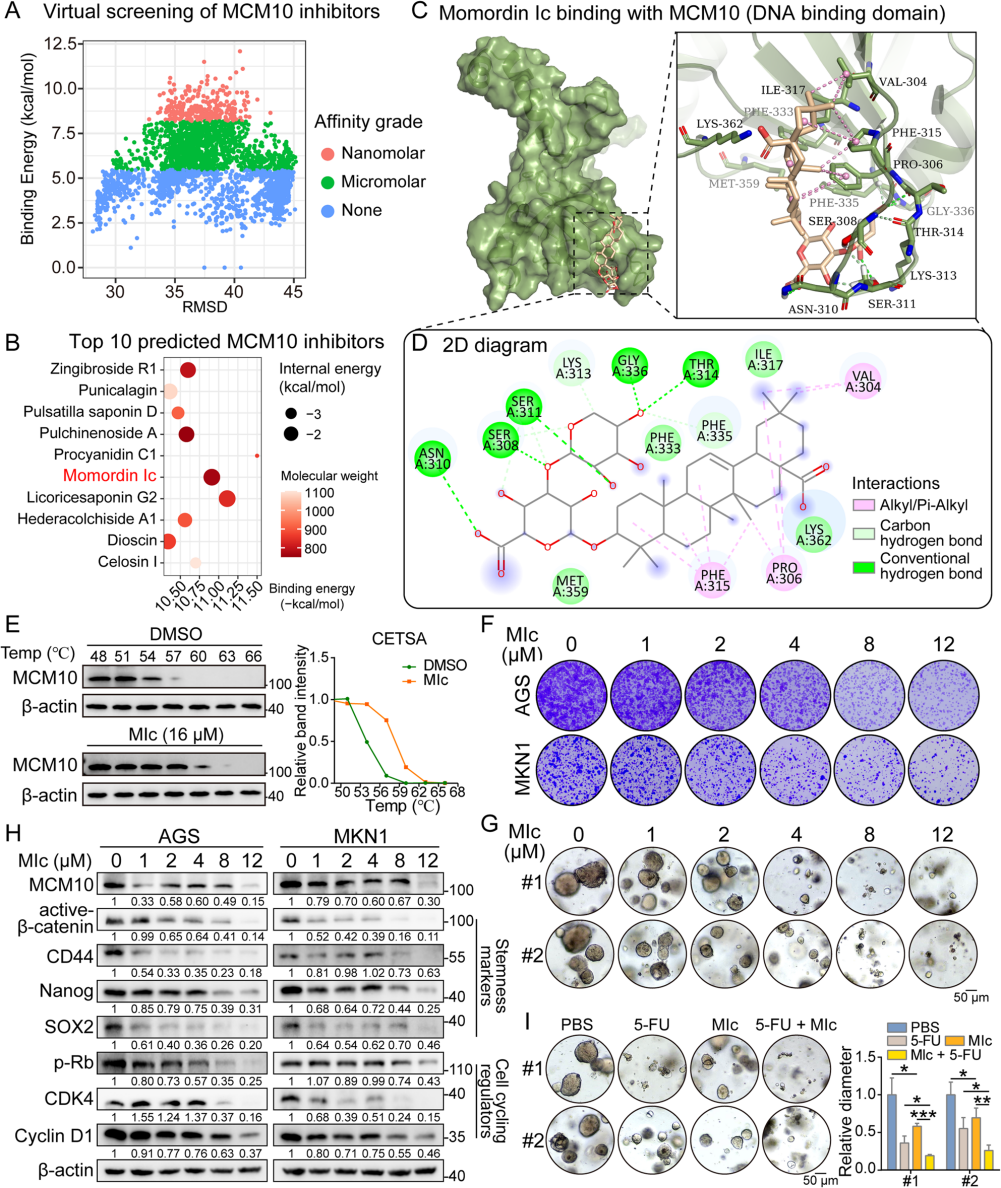
Momordin Ic (MIc) is identified as a promising MCM10-targeted inhibitor and demonstrated synergy with chemotherapy. **A**-**B** Through in silico screening of 2,725 natural compounds, MIc was selected as the top MCM10 inhibitor based on binding affinity and drug-likeness properties. **C**-**D** Predicted binding pattern and inter-molecular interactions between MIc and the DNA binding domain of MCM10. **E** CETSA confirmed the direct binding between MIc and the MCM10 protein. **F**-**G** Colony formation ability of GC cell lines (*n* = 3) and organoid growth (*n* = 5) were suppressed by MIc treatment in a dose-dependent manner. **H** Western blot analysis showed reduced expression of stemness markers and cell cycle regulators following MIc treatment. **I** MIc administration showed synergetic inhibition with 5-FU on GC organoid growth (*n* = 5). (*, *P* < 0.05; **, *P* < 0.01; ***, *P* < 0.001)

High-throughput virtual screening was conducted in a candidate library containing 2725 natural products, which are active ingredients of traditional Chinese medicine and with efficient bioactivity (Fig. 8A). The top 10 candidates were screened out ranked by binding affinity and referring to the root-mean-square deviation (RMSD) of binding conformation (Fig. 8B). Eventually, MIc was selected for its advantages in binding affinity with MCM10 DNA binding domain, molecular weight, internal stability, and the composition of intermolecular interactions (Fig. 8C-D). According to the principle of ligand-induced thermal stabilization, MIc was supposed to enhance the resistance against heat-induced denaturation of MCM10. Subsequent CETSA demonstrated substantial degradation of MCM10 when heated to 57 °C, whereas MIc treatment significantly enhanced the thermal stability of MCM10 (Fig. 8E). Functional assays indicated that MIc treatment suppressed colony formation in GC cells and inhibited the growth of organoids in a dose-dependent manner (Fig. 8F-G). Western blot analysis further revealed dose-dependent downregulation of stemness markers and cell cycle regulators following MIc treatment, confirming its inhibitory effect on MCM10 expression and related oncogenic processes, including DNA replication and stemness acquisition (Fig. 8H). Moreover, in GC organoid models, combined treatment with MIc and 5-FU exhibited enhanced anti-tumor efficacy compared to 5-FU monotherapy (Fig. 8I). In vivo data demonstrated that administration of MIc significantly impaired the growth of AGS-derived xenografts in NSG mice compared to the vehicle control group. Besides, tumors in the MIc + 5-FU group showed the most pronounced growth inhibition, supporting a synergistic effect and confirming that targeting MCM10 in vivo can overcome chemoresistance (Supplementary Fig. S13A). IHC staining results demonstrated significant MCM10 inhibition in CDXs harvested from MIc-administrated group (Supplementary Fig. S13B-C). As for the safety profiles of MIc treatment, H&E staining of the mouse’s major organs (heart, liver, spleen, lung, and kidney) revealed no evidence of severe morphological damage or overt toxicity (Supplementary Fig. S13D).

## Discussion

GC remains one of the most lethal malignancies worldwide, accounting for over 783,000 deaths in 2021 [2]. A primary cause of this high mortality is the development of drug resistance to systemic therapy, which promotes tumor recurrence and metastasis. CSCs are widely considered as key drivers of tumor heterogeneity and therapy resistance, partly due to their plasticity and differentiation capacity [38]. While understanding the molecular mechanisms regulating CSC self-renewal, drug resistance, and aggressiveness has become increasingly important [39], a significant gap remains in clinically viable inhibitors that specifically target CSC membrane proteins or stemness regulators in GC. Our findings demonstrated MCM10 as a critical molecular driver of this aggressive phenotype and established its direct inhibition as a validated therapeutic strategy.

In this study, comprehensive analyses revealed MCM10 to be a highly upregulated gene in chemo-resistant GC cells. We examined MCM10 as the most upregulated MCM family member and demonstrated its strong correlation with poor patient prognosis in GC. We further demonstrated MCM10 as a key driver of gastric tumorigenesis, by promoting cell cycle progression and DNA repair. Functionally, MCM10 was critical for maintaining cancer stemness in gastrointestinal tumors. In addition, MCM10 knockdown reduced cell self-renewal, proliferation, invasiveness, and spheroid formation ability in AGS and MNK1 cell lines, further evidence for the cancer-promoting role of MCM10. Mechanistically, we elucidated that MCM10 exhibited its oncogenic effects primarily through the Wnt/β-catenin pathway, which is essential for MCM10-mediated CSC self-renewal and chemoresistance. Specifically, we uncovered a novel MCM10-β-catenin regulatory axis, demonstrating that MCM10 deficiency inhibited nuclear translocation and transcriptional activity of β-catenin, leading to downregulation of key oncogenic targets. This provides crucial mechanistic insight into how MCM10 links DNA replication to stemness acquisition in GC. Moreover, we propose that MCM10 acts as a direct downstream transcriptional target of the YAP1/TEAD4 complex. Integrating this with our functional data on MCM10-driven Wnt/β-catenin signaling, our work reveals a previously unexplored molecular crosstalk between the Hippo/YAP1 and Wnt/β-catenin pathways in GC. Collectively, these significant findings offer a novel explanatory framework for treatment resistance in GC and identify promising therapeutic targets for future investigation.

MCM10 has long been recognized as an oncogene responsible for cancer progression and a significant prognostic marker in different cancers. Significantly higher expression of MCM10 is also observed in patients with different types of cancers, such as breast cancer, lung cancer, cervical cancer, and urothelial carcinoma [40–42]. High MCM10 expression has also been associated with poor prognosis in prostate, breast, and ovarian cancers [43–45]. In breast cancer patients, the BRCA2-MCM10 association would restrain replication fork progression upon DNA damage [46]. Notably, researchers demonstrated that MCM10 depletion selectively inhibited the proliferation of malignant cells while sparing normal cells [47]. Earlier genomic analysis revealed frequent *MCM10* mutations in early stage GC [48]. Although our TCGA data mining focused on amplification and overexpression rather than mutations, this prior observation suggests that genetic alteration of *MCM10* may be an early event in tumorigenesis, potentially initiating its dysregulation. Bi-allelic mutation of *MCM10* has been proven to trigger premature arrest during the differentiation of specific cardiac and immune cell lineages, leading to the clinical phenotypes of natural killer cell deficiency and restrictive cardiomyopathy [49, 50]. However, the implications of *MCM10* missense mutations or gene amplifications in oncogenesis remain poorly understood and represent an emerging area of investigation.

By securing genome duplication and mitigating replicative stress, MCM10 can create a permissive cellular state that supports the self-renewal and pluripotency signatures characteristic of cancer stem cells. As a critical replisome component, MCM10 ensures efficient DNA replication fork progression, directly sustaining proliferative capacity [51]. Furthermore, MCM10 stabilizes replication forks under stress, facilitating homologous recombination repair through interactions with proteins like RAD51 [52], thereby preventing catastrophic genomic instability. This preservation of genomic integrity is critical for maintaining the self-renewal capacity of stem-like cells. Additionally, MCM10 may influence stemness regulatory circuits by modulating the activity of core pluripotency factors, such as OCT4 or SOX2 [47]. MCM10 was also demonstrated to promote breast cancer cell migration and invasion via Wnt/β-catenin signaling [44]. A recent review comprehensively summarizes its diverse oncogenic functions across multiple cancer types, including roles in metastasis and therapy resistance [27]. In this study, we also demonstrate that MCM10 modulates β-catenin’s nuclear accumulation and transcriptional activity. IP-MS analysis identified HSPA8 as a prominent and specific binding partner of MCM10, which is known to prevent β-catenin degradation, thereby facilitating its nuclear translocation [53]. While this initial IP-MS did not detect a direct, stable interaction between MCM10 and β-catenin itself, the identification of HSPA8 provides a novel mechanistic hypothesis that appeals for further investigation. A recent study highlighted that MCM10 activation promotes glycolysis, thereby driving stemness and paclitaxel resistance in GC cells [54]. While we did not directly assess metabolic alterations, it is plausible that the replication stress and transcriptional rewiring orchestrated by the YAP1/TEAD4-MCM10 module create a cellular state that simultaneously activates glycolytic metabolism to support rapid proliferation and stemness maintenance.

By establishing TEAD4 as a key transcriptional regulator of MCM10, our study positioned the Hippo/YAP1 pathway as an upstream controller of the DNA replication licensing machinery. MCM10 is a critical and often limiting factor for the assembly and activation of the essential complex that unwinds DNA at replication origins [24, 55]. Studies in other systems showed that the dysregulation of replication licensing factors is a common strategy in cancer cells to promote unscheduled proliferation and genomic instability [56]. Consequently, by mediating MCM10 expression, TEAD4 can directly influence the fundamental process of DNA replication origin firing, thereby fueling the rampant proliferative capacity. Our data also revealed a valuable nuance between the correlation patterns in different public cohorts. The strong correlations in GC patient cohorts (TCGA, ACRG) underscore the physiological relevance of this axis in the complex TME. The relatively weaker correlation in the CCLE cell line database is informative, as cultured cell lines often acquire additional genetic and epigenetic alterations that can diversify their transcriptional dependencies beyond their original in vivo drivers. Furthermore, the correlation between MCM10 and YAP1 mRNA levels in the TCGA cohort is not as strong as that with TEAD4, which is consistent from a mechanistic perspective. TEAD4 is the stable DNA-binding component of the complex, and its expression level may more directly influence the transcriptional output of target genes. In contrast, YAP1 activity is primarily regulated post-translationally through its nucleo-cytoplasmic shuttling, making its mRNA level a less direct indicator of its functional nuclear activity.

Efforts to therapeutically target cancer stemness are constrained by the on-target toxicity of inhibiting master regulators, such as Wnt/β-catenin, Notch, and YAP1/TEADs, due to their essential roles in normal stem cell physiology. To improve the high failure rate of Phase II clinical trials, which often stems from unsuitable target selection, a successful target must demonstrate disease relevance, a favorable safety profile, and applicability to a defined patient population [57–59]. Our study identifies MCM10 as a YAP1-driven effector of chemotherapy resistance, offering a superior therapeutic alternative. Inhibiting MCM10 is predicted to minimize on-target toxicity compared to targeting the upstream Hippo/YAP1 pathway, which is vital for organ size, tissue homeostasis, and development [60]. This is supported by MCM10’s restricted normal tissue expression and the favorable preclinical safety of its inhibitor, MIc, a naturally derived, orally bioactive triterpenoid saponin isolated from *Kochia scoparia*. In vivo studies have demonstrated minimal toxicity of MIc treatment to major organs in mice, as well as its established therapeutic applications in inflammation and atherosclerosis [61, 62]. In hepatocellular carcinoma (HCC) patients, MCM10 has been identified as a promising target for counteracting sorafenib resistance by disrupting cancer stemness [63].

The identification of MCM10 as a direct downstream target of the YAP1/TEAD4 complex also provides a compelling rationale for its use as a predictive biomarker. Tumors with hyperactivated YAP1 signaling are functionally dependent on high MCM10 expression to drive their uncontrolled replication and maintain stemness-like properties. This dependency introduces a significant therapeutic vulnerability. Supported by promising pharmacokinetic results and a reliable safety profile of MIc, MCM10 prevails as a promising target. Consequently, the YAP1-MCM10 axis qualifies as a robust biomarker for patient stratification. In terms of sensitivity, tumors exhibiting YAP1 activation or high MCM10 expression are inherently more likely to respond to MCM10-targeted therapy. In terms of specificity, this biomarker can effectively exclude patients whose tumors proliferate via alternative, MCM10-independent pathways. This enrichment increases the likelihood of therapeutic benefit and enhances the potential for clinical success. To add up with, stratification of GC patients based on biomarkers, such as nuclear YAP1 overexpression, may be feasible for the treatment with MCM10 inhibitors. The application of biomarkers has been shown to correlate with increased success within the drug-development pipeline [64, 65].

While this study significantly advances the understanding of the role of MCM10 in GC by delineating its position within the YAP1/TEAD4-β-catenin oncogenic axis and its functional roles in regulating cancer cell stemness/chemoresistance acquisition, this work still has some limitations. Firstly, the proposed model wherein MCM10 interacts with HSPA8 to stabilize β-catenin requires rigorous biochemical validation to confirm the formation of a ternary complex, elucidate the specific interacting domains, and quantify its functional impact on β-catenin protein half-life and nuclear import kinetics. Secondly, the therapeutic potential of MIc as an MCM10-targeted agent warrants a more comprehensive preclinical evaluation, including a detailed assessment of its absorption, distribution, metabolism, excretion, and toxicity profile in relevant animal models, as well as a systematic investigation of its proteome-wide specificity to fully understand its mechanism of action and anticipate potential side effects. Thirdly, the study establishes MCM10 as a necessary effector in bridging cancer cell DNA replication and stemness acquisition, but a systems-level understanding of its broader transcriptional and functional network is lacking. Employing integrative multi-omics approaches could reveal a comprehensive regulon network, which would clarify whether MCM10 acts as a central hub coordinating multiple oncogenic programs and functions. Addressing these points will be crucial for translating these findings into a deeper mechanistic understanding and potential therapeutic strategies.

## Conclusion

In conclusion, this study demonstrated that MCM10 was upregulated in GC patients and associated with poor prognosis. Knockdown of MCM10 exerted anti-tumor effects by suppressing cell cycling progression, promoting DNA damage, and disrupting stemness maintenance in GC cells. The oncogenic role of MCM10 was also validated in PDO and CDX models. Mechanistically, MCM10 activates the Wnt/β-catenin signaling through promoting β-catenin nuclear accumulation and its transcriptional activity. Furthermore, we also validated MCM10 as a direct downstream effector of YAP1/TEAD4 transcription complex. Lastly, we also screened a small molecular inhibitor specifically targeting MCM10, which has been further validated with a potent inhibitory effect on MCM10 expression and awaits further optimization. Taken together, our study highlighted the oncogenic roles and deep mechanisms of MCM10 in gastric carcinogenesis and the therapeutic potential of targeting this critical juncture of Hippo and Wnt signaling transduction.

## Supplementary Information

Supplementary Material 1

Supplementary Material 2

## Data Availability

The datasets analyzed during the current study are available from the corresponding author upon reasonable request.

## Abbreviations

5-FU: 5-Fluorouracil
ACRG: Asian Cancer Research Group
AEEC: Animal Experimentation Ethics Committee
ATCC: American Type Culture Collection
BrdU: Bromodeoxyuridine
BSA: Bovine Serum Albumin
CCLE: Cancer Cell Line Encyclopedia
CDX: Cell-derived xenograft
CETSA: Cellular thermal shift assay
ChIP: Chromatin immunoprecipitation
CMG: Cdc45-MCM-GINS
CSC: Cancer stem cell
CUHK: The Chinese University of Hong Kong
DEGs: Differentially expressed genes
EDTA: Ethylenediaminetetraacetic acid
FBS: Fetal bovine serum
GC: Gastric cancer
GDSC: Genomics of Drug Sensitivity in Cancer
GSEA: Gene Set Enrichment Analysis
GSVA: Gene Set Variation Analysis
HCC: Hepatocellular carcinoma
IHC: Immunohistochemistry
IF: Immunofluorescence
MIc: Momordin Ic
MCM: Minichromosome maintenance
MNNG: Methylnitronitrosoguanidine
MTT: 3-(4,5-Dimethylthiazol-2-yl)-2,5-Diphenyltetrazolium Bromide (cell viability assay)
NSG mice: NOD scid gamma mice
PBS: Phosphate-buffered saline
PDB: Protein Data Bank
PDO: Patient-derived organoid
PFA: Paraformaldehyde
qRT-PCR: Quantitative real-time polymerase chain reaction
RPMI: Roswell Park Memorial Institute (culture medium)
RMSD: Root-mean-square deviation
scRNA-seq: Single-cell RNA sequencing
shRNA: Short hairpin RNA
siRNA: Small interfering RNA
TCGA-STAD: The Cancer Genome Atlas-Stomach Adenocarcinoma
TME: Tumor microenvironment
TEADs: TEA domain transcription factors
TSS: Transcription start site
UMAP: Uniform manifold approximation and projection
YAP1: Yes-associated protein 1

## References

[1] Sundar R, Nakayama I, Markar SR, Shitara K, van Laarhoven HWM, Janjigian YY, Smyth EC. Gastric cancer. Lancet. 2025;405:2087–102. 40319897 10.1016/S0140-6736(25)00052-2

[2] Bray F, Laversanne M, Sung H, Ferlay J, Siegel RL, Soerjomataram I, Jemal A. Global cancer statistics 2022: GLOBOCAN estimates of incidence and mortality worldwide for 36 cancers in 185 countries. CA Cancer J Clin. 2024;74:229–63. 38572751 10.3322/caac.21834

[3] Guan WL, He Y, Xu RH. Gastric cancer treatment: recent progress and future perspectives. J Hematol Oncol. 2023;16:57. 37245017 10.1186/s13045-023-01451-3 PMC10225110

[4] Che G, Yin J, Wang W, Luo Y, Chen Y, Yu X, Wang H, Liu X, Chen Z, Wang X, et al. Circumventing drug resistance in gastric cancer: A spatial multi-omics exploration of chemo and immuno-therapeutic response dynamics. Drug Resist Updat. 2024;74:101080. 38579635 10.1016/j.drup.2024.101080

[5] Kim R, An M, Lee H, Mehta A, Heo YJ, Kim KM, Lee SY, Moon J, Kim ST, Min BH, et al. Early Tumor-Immune Microenvironmental Remodeling and Response to First-Line Fluoropyrimidine and Platinum Chemotherapy in Advanced Gastric Cancer. Cancer Discov. 2022;12:984–1001. 34933901 10.1158/2159-8290.CD-21-0888 PMC9387589

[6] Marin JJ, Al-Abdulla R, Lozano E, Briz O, Bujanda L, Banales JM, Macias RI. Mechanisms of Resistance to Chemotherapy in Gastric Cancer. Anticancer Agents Med Chem. 2016;16:318–34. 26234359 10.2174/1871520615666150803125121

[7] Longley DB, Harkin DP, Johnston PG. 5-fluorouracil: mechanisms of action and clinical strategies. Nat Rev Cancer. 2003;3:330–8. 12724731 10.1038/nrc1074

[8] Rao X, Zhang C, Luo H, Zhang J, Zhuang Z, Liang Z, Wu X. Targeting Gastric Cancer Stem Cells to Enhance Treatment Response. Cells 2022;11:2828. 10.3390/cells11182828 PMC9496718 36139403

[9] Cao L, Weng K, Li L, Lin G, Zhao Y, Gao Y, Huang X, Chen Q, Wang J, Zheng C, et al. BATF2 inhibits the stem cell-like properties and chemoresistance of gastric cancer cells through PTEN/AKT/β-catenin pathway. Theranostics. 2024;14:7007–22. 39629124 10.7150/thno.98389 PMC11610130

[10] Lu L, Wu M, Sun L, Li W, Fu W, Zhang X, Liu T. Clinicopathological and prognostic significance of cancer stem cell markers CD44 and CD133 in patients with gastric cancer: A comprehensive meta-analysis with 4729 patients involved. Med (Baltim). 2016;95:e5163. 10.1097/MD.0000000000005163 PMC5079331 27759647

[11] Lu T, Sun L, Zhu X. Yes-associated protein enhances proliferation and attenuates sensitivity to cisplatin in human gastric cancer cells. Biomed Pharmacother. 2018;105:1269–75. 30021363 10.1016/j.biopha.2018.06.031

[12] Messina B, Lo Sardo F, Scalera S, Memeo L, Colarossi C, Mare M, Blandino G, Ciliberto G, Maugeri-Saccà M, Bon G. Hippo pathway dysregulation in gastric cancer: from Helicobacter pylori infection to tumor promotion and progression. Cell Death Dis. 2023;14:21. 36635265 10.1038/s41419-023-05568-8 PMC9837097

[13] Zheng Y, Pan D. The Hippo Signaling Pathway in Development and Disease. Dev Cell. 2019;50:264–82. 31386861 10.1016/j.devcel.2019.06.003 PMC6748048

[14] Zhou Y, Huang T, Zhang J, Wong CC, Zhang B, Dong Y, Wu F, Tong JHM, Wu WKK, Cheng ASL, et al. TEAD1/4 exerts oncogenic role and is negatively regulated by miR-4269 in gastric tumorigenesis. Oncogene. 2017;36:6518–30. 28759040 10.1038/onc.2017.257 PMC5702719

[15] Ajani JA, Xu Y, Huo L, Wang R, Li Y, Wang Y, Pizzi MP, Scott A, Harada K, Ma L, et al. YAP1 mediates gastric adenocarcinoma peritoneal metastases that are attenuated by YAP1 inhibition. Gut. 2021;70:55–66. 32345613 10.1136/gutjnl-2019-319748 PMC9832914

[16] Song S, Wang Z, Li Y, Ma L, Jin J, Scott AW, Xu Y, Estrella JS, Song Y, Liu B, et al. PPARδ Interacts with the Hippo Coactivator YAP1 to Promote SOX9 Expression and Gastric Cancer Progression. Mol Cancer Res. 2020;18:390–402. 31796534 10.1158/1541-7786.MCR-19-0895

[17] Giraud J, Molina-Castro S, Seeneevassen L, Sifré E, Izotte J, Tiffon C, Staedel C, Boeuf H, Fernandez S, Barthelemy P, et al. Verteporfin targeting YAP1/TAZ-TEAD transcriptional activity inhibits the tumorigenic properties of gastric cancer stem cells. Int J Cancer. 2020;146:2255–67. 31489619 10.1002/ijc.32667

[18] Lv G, Wang Q, Lin L, Ye Q, Li X, Zhou Q, Kong X, Deng H, You F, Chen H, et al. mTORC2-driven chromatin cGAS mediates chemoresistance through epigenetic reprogramming in colorectal cancer. Nat Cell Biol. 2024;26:1585–96. 39080411 10.1038/s41556-024-01473-0 PMC11392818

[19] Patel SM, Dash RC, Hadden MK. Translesion synthesis inhibitors as a new class of cancer chemotherapeutics. Expert Opin Investig Drugs. 2021;30:13–24. 33179552 10.1080/13543784.2021.1850692 PMC7832080

[20] Thompson R, Meuth M, Woll P, Zhu Y, Danson S. Treatment with the Chk1 inhibitor Gö6976 enhances cisplatin cytotoxicity in SCLC cells. Int J Oncol. 2012;40:194–202. 21894433 10.3892/ijo.2011.1187

[21] Wang Y, Chen H, Zhang J, Cheng ASL, Yu J, To KF, Kang W. MCM family in gastrointestinal cancer and other malignancies: From functional characterization to clinical implication. Biochim Biophys Acta Rev Cancer. 2020;1874:188415. 32822825 10.1016/j.bbcan.2020.188415

[22] Zhao X, Wang J, Jin D, Cheng J, Chen H, Li Z, Wang Y, Lou H, Zhu JK, Du X, Gong Z. AtMCM10 promotes DNA replication-coupled nucleosome assembly in Arabidopsis. J Integr Plant Biol. 2023;65:203–22. 36541721 10.1111/jipb.13438

[23] Baxley RM, Bielinsky AK. Mcm10: A Dynamic Scaffold at Eukaryotic Replication Forks. Genes (Basel) 2017;8:73. 10.3390/genes8020073 PMC5333062 28218679

[24] Kanke M, Kodama Y, Takahashi TS, Nakagawa T, Masukata H. Mcm10 plays an essential role in origin DNA unwinding after loading of the CMG components. Embo j. 2012;31:2182–94. 22433840 10.1038/emboj.2012.68 PMC3343466

[25] Masnovo C, Paleiov Z, Dovrat D, Baxter LK, Movafaghi S, Aharoni A, Mirkin SM. Stabilization of expandable DNA repeats by the replication factor Mcm10 promotes cell viability. Nat Commun. 2024;15:10532. 39627228 10.1038/s41467-024-54977-6 PMC11615337

[26] Izumi M, Yatagai F, Hanaoka F. Localization of human Mcm10 is spatially and temporally regulated during the S phase. J Biol Chem. 2004;279:32569–77. 15136575 10.1074/jbc.M314017200

[27] Ahmed SMQ, Sasikumar J, Laha S, Das SP. Multifaceted role of the DNA replication protein MCM10 in maintaining genome stability and its implication in human diseases. Cancer Metastasis Rev. 2024;43:1353–71. 39240414 10.1007/s10555-024-10209-3

[28] Xie F, Lyu Y, Chen B, Leung HW, Yu P, Feng T, Fang C, Cheung AHK, Zhou B, Jiang J, et al. STK3 is a transcriptional target of YAP1 and a hub component in the crosstalk between Hippo and Wnt signaling pathways during gastric carcinogenesis. Mol Cancer. 2025;24:186. 40604818 10.1186/s12943-025-02391-x PMC12220525

[29] Chen C, Wang Z, Lin Q, Li M, Xu L, Fu Y, Zhao X, Ma Z, Xu J, Zhou S, et al. NAT10 Promotes Gastric Cancer Liver Metastasis by Modulation of M2 Macrophage Polarization and Metastatic Tumor Cell Hepatic Adhesion. Adv Sci (Weinh). 2025;12:e2410263. 39985269 10.1002/advs.202410263 PMC12005778

[30] Comprehensive molecular characterization of gastric adenocarcinoma. Nature. 2014;513:202–9. 25079317 10.1038/nature13480 PMC4170219

[31] Cristescu R, Lee J, Nebozhyn M, Kim KM, Ting JC, Wong SS, Liu J, Yue YG, Wang J, Yu K, et al. Molecular analysis of gastric cancer identifies subtypes associated with distinct clinical outcomes. Nat Med. 2015;21:449–56. 25894828 10.1038/nm.3850

[32] Yang W, Soares J, Greninger P, Edelman EJ, Lightfoot H, Forbes S, Bindal N, Beare D, Smith JA, Thompson IR, et al. Genomics of Drug Sensitivity in Cancer (GDSC): a resource for therapeutic biomarker discovery in cancer cells. Nucleic Acids Res. 2013;41:D955–961. 23180760 10.1093/nar/gks1111 PMC3531057

[33] Mora-Lagos B, Cartas-Espinel I, Riquelme I, Parker AC, Piccolo SR, Viscarra T, Reyes ME, Zanella L, Buchegger K, Ili C, Brebi P. Functional and transcriptomic characterization of cisplatin-resistant AGS and MKN-28 gastric cancer cell lines. PLoS ONE. 2020;15:e0228331. 31990955 10.1371/journal.pone.0228331 PMC6986722

[34] Sathe A, Grimes SM, Lau BT, Chen J, Suarez C, Huang RJ, Poultsides G, Ji HP. Single-Cell Genomic Characterization Reveals the Cellular Reprogramming of the Gastric Tumor Microenvironment. Clin Cancer Res. 2020;26:2640–53. 32060101 10.1158/1078-0432.CCR-19-3231 PMC7269843

[35] Kumar V, Ramnarayanan K, Sundar R, Padmanabhan N, Srivastava S, Koiwa M, Yasuda T, Koh V, Huang KK, Tay ST, et al. Single-Cell Atlas of Lineage States, Tumor Microenvironment, and Subtype-Specific Expression Programs in Gastric Cancer. Cancer Discov. 2022;12:670–91. 34642171 10.1158/2159-8290.CD-21-0683 PMC9394383

[36] Du W, Stauffer ME, Eichman BF. Structural biology of replication initiation factor Mcm10. Subcell Biochem. 2012;62:197–216. 22918587 10.1007/978-94-007-4572-8_11 PMC5023279

[37] Tang Y, Fang G, Guo F, Zhang H, Chen X, An L, Chen M, Zhou L, Wang W, Ye T, et al. Selective Inhibition of STRN3-Containing PP2A Phosphatase Restores Hippo Tumor-Suppressor Activity in Gastric Cancer. Cancer Cell. 2020;38:115–e128119. 32589942 10.1016/j.ccell.2020.05.019

[38] Dagogo-Jack I, Shaw AT. Tumour heterogeneity and resistance to cancer therapies. Nat Rev Clin Oncol. 2018;15:81–94. 29115304 10.1038/nrclinonc.2017.166

[39] Huang T, Song X, Xu D, Tiek D, Goenka A, Wu B, Sastry N, Hu B, Cheng SY. Stem cell programs in cancer initiation, progression, and therapy resistance. Theranostics. 2020;10:8721–43. 32754274 10.7150/thno.41648 PMC7392012

[40] Mughal MJ, Mahadevappa R, Kwok HF. DNA replication licensing proteins: Saints and sinners in cancer. Semin Cancer Biol. 2019;58:11–21. 30502375 10.1016/j.semcancer.2018.11.009

[41] Mahadevappa R, Neves H, Yuen SM, Jameel M, Bai Y, Yuen HF, Zhang SD, Zhu Y, Lin Y, Kwok HF. DNA Replication Licensing Protein MCM10 Promotes Tumor Progression and Is a Novel Prognostic Biomarker and Potential Therapeutic Target in Breast Cancer. Cancers (Basel) 2018;10:282. 10.3390/cancers10090282 PMC6162382 30135378

[42] Ahmed SMQ, Laha S, Das R, Ifthikar MA, Das SP. MCM10 expression is linked to cervical cancer aggressiveness. Front Mol Med. 2023;3:1009903. 39086679 10.3389/fmmed.2023.1009903 PMC11285692

[43] Cui F, Hu J, Ning S, Tan J, Tang H. Overexpression of MCM10 promotes cell proliferation and predicts poor prognosis in prostate cancer. Prostate. 2018;78:1299–310. 30095171 10.1002/pros.23703 PMC6282949

[44] Yang WD, Wang L. MCM10 facilitates the invaded/migrated potentials of breast cancer cells via Wnt/β-catenin signaling and is positively interlinked with poor prognosis in breast carcinoma. J Biochem Mol Toxicol. 2019;33:e22330. 30990947 10.1002/jbt.22330

[45] Wu Z, Wang Y, Li J, Wang H, Tuo X, Zheng J. MCM10 is a Prognostic Biomarker and Correlated With Immune Checkpoints in Ovarian Cancer. Front Genet. 2022;13:864578. 35664337 10.3389/fgene.2022.864578 PMC9161093

[46] Kang Z, Fu P, Alcivar AL, Fu H, Redon C, Foo TK, Zuo Y, Ye C, Baxley R, Madireddy A, et al. BRCA2 associates with MCM10 to suppress PRIMPOL-mediated repriming and single-stranded gap formation after DNA damage. Nat Commun. 2021;12:5966. 34645815 10.1038/s41467-021-26227-6 PMC8514439

[47] Murayama T, Takeuchi Y, Yamawaki K, Natsume T, Li M, Marcela RN, Nishimura T, Kogure Y, Nakata A, Tominaga K, et al. MCM10 compensates for Myc-induced DNA replication stress in breast cancer stem-like cells. Cancer Sci. 2021;112:1209–24. 33340428 10.1111/cas.14776 PMC7935783

[48] Kang G, Hwang WC, Do IG, Wang K, Kang SY, Lee J, Park SH, Park JO, Kang WK, Jang J, et al. Exome sequencing identifies early gastric carcinoma as an early stage of advanced gastric cancer. PLoS ONE. 2013;8:e82770. 24376576 10.1371/journal.pone.0082770 PMC3871845

[49] Baxley RM, Leung W, Schmit MM, Matson JP, Yin L, Oram MK, Wang L, Taylor J, Hedberg J, Rogers CB, et al. Bi-allelic MCM10 variants associated with immune dysfunction and cardiomyopathy cause telomere shortening. Nat Commun. 2021;12:1626. 33712616 10.1038/s41467-021-21878-x PMC7955084

[50] Mace EM, Paust S, Conte MI, Baxley RM, Schmit MM, Patil SL, Guilz NC, Mukherjee M, Pezzi AE, Chmielowiec J, et al. Human NK cell deficiency as a result of biallelic mutations in MCM10. J Clin Invest. 2020;130:5272–86. 32865517 10.1172/JCI134966 PMC7524476

[51] Brosh RM Jr., Trakselis MA. Fine-tuning of the replisome: Mcm10 regulates fork progression and regression. Cell Cycle. 2019;18:1047–55. 31014174 10.1080/15384101.2019.1609833 PMC6592251

[52] Alver RC, Zhang T, Josephrajan A, Fultz BL, Hendrix CJ, Das-Bradoo S, Bielinsky AK. The N-terminus of Mcm10 is important for interaction with the 9-1-1 clamp and in resistance to DNA damage. Nucleic Acids Res. 2014;42:8389–404. 24972833 10.1093/nar/gku479 PMC4117747

[53] Yang K, Liu Z, Wang H, Xiao Z, Zhao W, Gong W. Microbial metabolite trimethylamine-N-oxide facilitates colorectal inflammation-cancer transformation by blocking lysosomal degradation of Wnt signaling. Gut Microbes. 2025;17:2597626. 41376600 10.1080/19490976.2025.2597626 PMC12710936

[54] Wu Z, Fang Y, Wu J, Wang J, Ling Y, Liu T, Tong Q, Yao Y. Activation of Glycolysis by MCM10 Increases Stemness and Paclitaxel Resistance in Gastric Cancer Cells. Turk J Gastroenterol. 2023;34:1107–15. 37860833 10.5152/tjg.2023.23169 PMC10724805

[55] Watase G, Takisawa H, Kanemaki MT. Mcm10 plays a role in functioning of the eukaryotic replicative DNA helicase, Cdc45-Mcm-GINS. Curr Biol. 2012;22:343–9. 22285032 10.1016/j.cub.2012.01.023

[56] Macheret M, Halazonetis TD. DNA replication stress as a hallmark of cancer. Annu Rev Pathol. 2015;10:425–48. 25621662 10.1146/annurev-pathol-012414-040424

[57] Vandana JJ, Manrique C, Lacko LA, Chen S. Human pluripotent-stem-cell-derived organoids for drug discovery and evaluation. Cell Stem Cell. 2023;30:571–91. 37146581 10.1016/j.stem.2023.04.011 PMC10775018

[58] Lordick F, Shitara K, Janjigian YY. New agents on the horizon in gastric cancer. Ann Oncol. 2017;28:1767–75. 28184417 10.1093/annonc/mdx051

[59] Brungs D, Aghmesheh M, Vine KL, Becker TM, Carolan MG, Ranson M. Gastric cancer stem cells: evidence, potential markers, and clinical implications. J Gastroenterol. 2016;51:313–26. 26428661 10.1007/s00535-015-1125-5

[60] Fu M, Hu Y, Lan T, Guan KL, Luo T, Luo M. The Hippo signalling pathway and its implications in human health and diseases. Signal Transduct Target Ther. 2022;7:376. 36347846 10.1038/s41392-022-01191-9 PMC9643504

[61] Han LK, Nose R, Li W, Gong XJ, Zheng YN, Yoshikawa M, Koike K, Nikaido T, Okuda H, Kimura Y. Reduction of fat storage in mice fed a high-fat diet long term by treatment with saponins prepared from Kochia scoparia fruit. Phytother Res. 2006;20:877–82. 16892459 10.1002/ptr.1981

[62] Yadav VR, Prasad S, Sung B, Kannappan R, Aggarwal BB. Targeting inflammatory pathways by triterpenoids for prevention and treatment of cancer. Toxins (Basel). 2010;2:2428–66. 22069560 10.3390/toxins2102428 PMC3153165

[63] Zhang Z, Liang L, Li Y, Fan Y, Liu Y, He Z, Wu X, Cong L, Jiang Y, Wan T. Targeting MCM10 disrupts cancer stemness and counteracts sorafenib resistance in hepatocellular carcinoma. Cancer Gene Ther. 2025;32:1076–89. 40750706 10.1038/s41417-025-00946-0 PMC12535913

[64] Cleary JM, Aguirre AJ, Shapiro GI, D’Andrea AD. Biomarker-Guided Development of DNA Repair Inhibitors. Mol Cell. 2020;78:1070–85. 32459988 10.1016/j.molcel.2020.04.035 PMC7316088

[65] Passaro A, Al Bakir M, Hamilton EG, Diehn M, André F, Roy-Chowdhuri S, Mountzios G, Wistuba II, Swanton C, Peters S. Cancer biomarkers: Emerging trends and clinical implications for personalized treatment. Cell. 2024;187:1617–35. 38552610 10.1016/j.cell.2024.02.041 PMC7616034

