# Supplementary material for: MCM10, a novel YAP1/TEAD4 target, drives gastric cancer progression by bridging DNA replication to stemness acquisition
Source: Mol Cancer. 2026 Feb 27;25:87. doi: 10.1186/s12943-026-02623-8 (PMC13049980; doi:10.1186/s12943-026-02623-8)
Supplement: Supplementary file 1 — Supplementary Material 1 [file 12943_2026_2623_MOESM1_ESM.docx]

Supplementary Materials for

**MCM10, a novel YAP1/TEAD4 target, drives gastric cancer progression by bridging DNA replication to stemness acquisition**

Fuda Xie^1,2,3,#^, Hoi Wing Leung^1,#^, Yang Lyu^1,#^, Peiyao Yu^1^, Tiejun Feng^1^, Bonan Chen^1,2,3^, Jialin Wu^1^, Jenson Tham^1^, Canbin Fang^1^, Alvin H.K. Cheung^1^, Chit Chow^1^, Jianhui Jiang^4^, Jintao Hu^5^, Fengbin Zhang^6^, Chaowei Zhu^7^, Keli Zhong^7^, Meiheng Sun^8^, Ge Zhang^8^, Sifan Yu^8^, Dazhi Xu^9^, Shouyu Wang^10^, Bing Huang^11^, Kangmin Zhuang^11^, Xiaobei Luo^11^, Aimin Li^11^, Qing Guo^12^, Chanchan Gao^13^, Bin Zhang^14^, Yuan Ma^15^, William KK Wu^16^, Liwei An^17^, Chi Chun Wong^2,18^, Jun Yu^2,18^, Ka Fai To^1^, Wei Kang^1,2,3,§^

§ Corresponding author: Wei Kang (E-mail: weikang@cuhk.edu.hk)

**This file includes:**

Supplementary Materials and Methods

Supplementary Figure S1-S13

***In vitro* functional assays**

For MTT cell viability assay, cells were seeded at 1000 cells per well with 5 replicates for each group (MCM10 siRNA and scramble siRNA control). Absorbance was measured at 575 nm (Victor3, Perken Elmer, Waltham). For colony formation assay, cells were seeded in 6-well plates overnight, and cells were transfected in triplicates for each group. Following 6 days post-transfection, the colonies were fixed with 70% ethanol and stained in 1.5% crystal violet. Colonies with more than 50 cells were counted. For cell invasion experiments, Matrigel-precoated Boyden chambers (BD Biosciences, #354480) were used. Transfected cells were resuspended in FBS-free medium, and a suspension of 5 × 10^4^ cells was seeded into the upper chamber of each transwell. The lower chamber was filled with medium supplemented with 10% (v/v) FBS. Following 24 hours incubation, the cells were fixed with methanol and stained with 1% Toluidine blue. Non-invading cells on the upper surface of the membrane were gently scraped using a cotton swab. For spheroid formation assays, GC cells were seeded at a density of 1,000 cells/well in 24-well ultra-low attachment plates. Cells were cultured in serum-free Advanced DMEM/F-12 (Life technologies), supplemented with B27 (1:50, Life technologies), 50 ng/mL EGF (Life technologies), 100 ng/mL FGF10 (Life technologies) and 1% penicillin/streptomycin (Gibco). Spheroid size was assessed based on diameter measurement taken 14 days after spheroid formation.

**RNA extraction and qRT-PCR**

The total RNA was extracted with TRIzol reagent (Invitrogen, Carlsbad, CA). cDNA synthesis was achieved with a High-Capacity cDNA Reverse Transcription Kits (Applied Biosystems, Carlsbad, CA). PCR experiments were performed using the 7500 Fast Real-Time PCR system (Applied Biosystems). Relative expression differences were calculated using the 2-ΔΔCT method with reference to GAPDH.

**Chromatin immunoprecipitation (ChIP) assay**

Cells were processed for chromatin immunoprecipitation (ChIP) assays according to the manufacturer's protocol (Cell Signaling Technology). Briefly, cells were cross-linked for 10 minutes with 1% formaldehyde, and lysed. Lysate pellets were resuspended and enzymatically digested with micrococcal nuclease for 15 - 20 minutes. Protein-DNA complexes were immunoprecipitated using TEAD4 antibody (Abcam, #58310) or Normal Rabbit IgG (Cell Signaling technology) bound to magnetic beads, eluted, and digested with proteinase K. For qPCR analysis of the ChIP DNA samples before amplicon generation, purified immunoprecipitates were dissolved in 50 μL of water. Standard PCR reactions using 2 μL of the immunoprecipitated DNA were performed with a SYBR Green PCR Kit (Applied Biosystems). The MCM10 promoter region in the TEAD4–precipitated chromatin was amplified by qPCR primers (forward primer sequence AAAGTGGCCATTTGGTCCATCA and reverse primer sequence CGTCAGGGCTGAAGAGAACTT. The amplicon is at -611 bp downstream of transcription start site (TSS). Calculation of TEAD4 occupancy on the MCM10 promoter was performed according to the ChIP-qPCR primer assay data analysis template from Sigma-Aldrich.

**Protein extraction and Western blot**

Protein extraction and Western blot analysis were performed as described in our previous studies [1]. Briefly, whole cell lysates were extracted using RIPA lysis buffer, and total protein was quantified with the BCA Protein Assay Kit (ThermoFisher Scientific). Proteins were resolved by 10% SDS polyacrylamide gel and transferred to PVDF membrane. The blots were blocked in 5% BSA in Tris-buffered saline Tween 20 (TBST) for 2 hours, and subsequently incubated with specific primary antibodies overnight at 4°C. After washing with TBST, the blots were incubated with HRP-conjugated secondary antibodies for 1 hour. The signal on the blots was visualized using enhanced chemiluminescence. Three independent biological replicates were conducted for all representing assays.

**BrdU Cell Proliferation Assay**

GC cells at the cell density of 3,000 cells/well were seeded in 96-well culture plate. The proliferative response was determined using a bromodeoxyuridine (BrdU) cell proliferation enzyme-linked immunosorbent assay (ELISA) kit (Beyotime, #C0071S), following the manufacturer’s instructions. The absorbance was measured at 485/535 nm using a microplate reader.

**β-catenin TCF binding luciferase reporter assay**

β-catenin transcriptional activity was assessed using a TCF/LEF-dependent luciferase reporter assay of (TOP/FOPFLASH system) [2]. Transfections were performed using Lipofectamine 2000 Reagent. Luciferase activities were measured 48 hours post-transfection using the Dual-Luciferase® Reporter Assay System (Promega), following the manufacturer’s protocol. Results are expressed as the ratio of firefly/Renilla luciferase activity.

**Comet assay**

To quantitatively assess the level of DNA strand breaks resulting from MCM10 depletion and/or chemotherapeutic treatment, a comet assay was performed. GC cells transfected with siCtrl or siMCM10 were treated with or without 5-FU for 24 or 48 hours. The comet assay was performed according to the manufacturer's instructions (Beyotime, # C2041M). Briefly, the treated cells were suspended in pre-warmed 0.7% low-melting-point agarose and immediately pipetted onto slides pre-coated with 1% normal-melting-point agarose. The cells were lysed overnight at 4℃ and subjected to electrophoresis. The samples were subsequently stained with propidium iodide (PI) and analyzed using a fluorescence microscope, where the tail moment and intensity are proportional to the amount of DNA damage.

**Immunoprecipitation-mass spectrometry (IP-MS) analysis**

Human recombinant his-tagged MCM10 protein (Antibodies, #ABIN7563621) based pull-down assay was performed and human serum albumin (HSA) was used as control. The obtained proteins were separated into 10% SDS-PAGE gel flowed by silver staining. The excised gel bands are subjected to in-gel digestion, where proteins are reduced, alkylated, and digested into peptides by trypsin. The peptides were separated using the UltiMate 3000 nano ultra-high performance liquid phase system. Peptides were analyzed by Thermo Scientific Q-Exactive mass spectrometry. The ion source voltage was set to 1.8 kV, and both the peptide precursor and its secondary fragments were detected and analyzed using a high-resolution Orbitrap. The primary mass spectrometry scanning range is set to 350-2000 Da, and the scan resolution is set to 70,000. The data acquisition mode uses a data-dependent scan procedure, in which the top 20 precursors of the highest signal intensity are selected after the primary scan to enter the HCD collision cell in turn for 28% use. The fragmentation energy is also performed for secondary mass spectrometry analysis. The secondary mass spectrometry scanning range relies on the primary precursor mass-to-charge ratio automatic selection, with a resolution set to 17,500.

**Cellular thermal shift assay (CETSA)**

The harvested cells were subjected to freeze-thaw cycles three times using liquid nitrogen to achieve complete lysis. The resulting cell lysate supernatants were then centrifuged at 20,000 g for 20 minutes at 4°C, diluted in PBS, and split into a Treated group (mixed with 10 μM MIc) and a Control group (mixed with an empty vehicle). After incubation for 30 minutes at room temperature, the lysates were divided into 50 μL aliquots, which were heated to the designated temperatures for 3 minutes using a thermal cycler (Applied Biosystems). The heated lysates were subsequently centrifuged at 20,000 g for 20 minutes at 4°C to collect supernatants containing the soluble protein fraction for western blot analysis.

**Supplementary Figure S1**


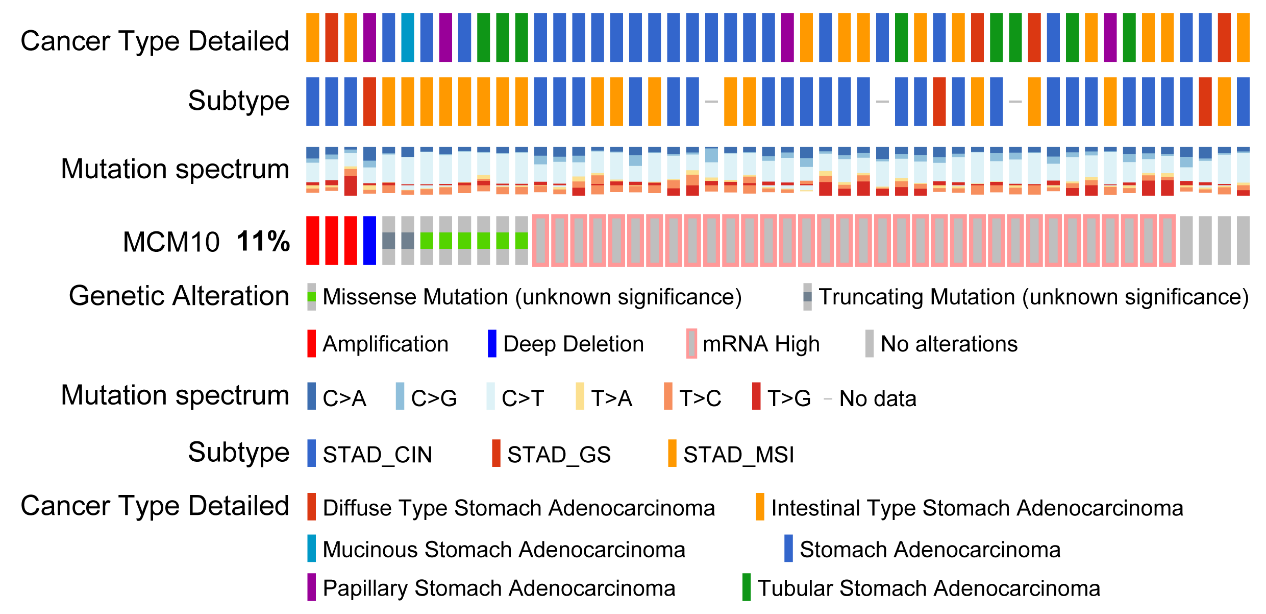


**Supplementary Figure S1** The genetic alterations of MCM10 in TCGA cohort. A case is classified as “mRNA High” if the *MCM10* mRNA expression achieves a Z-score greater than 2 relative to the distribution of expression in normal tissue samples (z = (x - μ) / σ, where x is the logarithmically transformed mRNA expression value in the case, and μ and σ are the mean and standard deviation of expression in all adjacent normal tissue samples in this cohort)

**Supplementary Figure S2**


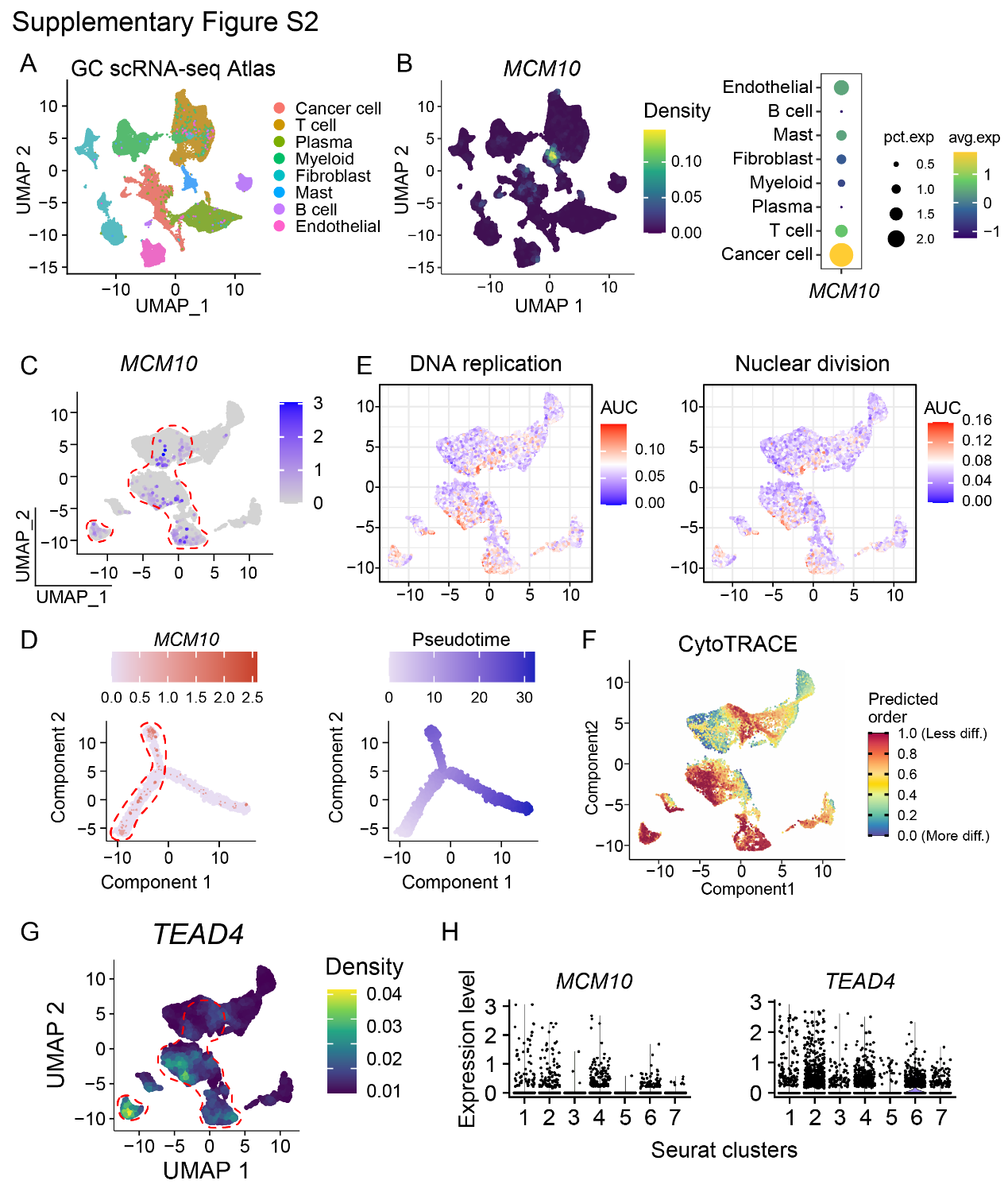


**Supplementary Figure S2** Independent dataset validating the key findings generated from scRNA-seq analysis. **(A-B)** *MCM10* expression is specifically enriched in malignant epithelial cell clusters. **(C-D)** Pseudotime analysis demonstrates *MCM10+* cancer cells are enriched in early phase of cell cycle process. **(E)** GSVA results show positive correlation with cell cycle activity signatures. **(G-H)** *MCM10+* cells exhibit high expression level of *TEAD4*.

**Supplementary Figure S3**


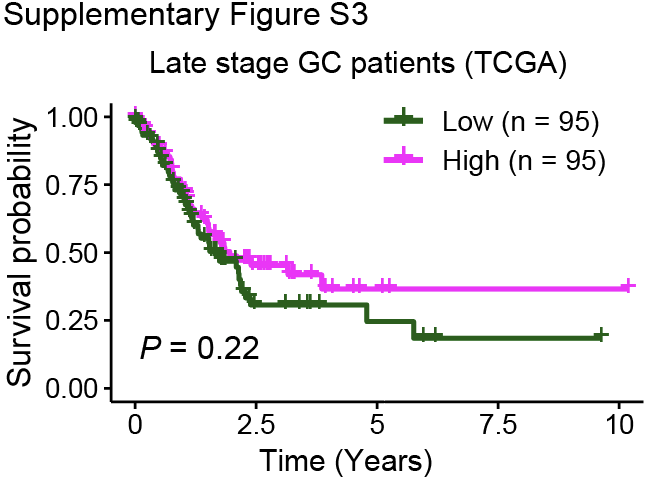


**Supplementary Figure S3** Survival analysis shows that high *MCM10* mRNA expression is not a poor prognosis predictor in late stage (Stage III and IV) GC patients.

**Supplementary Figure S4**

**
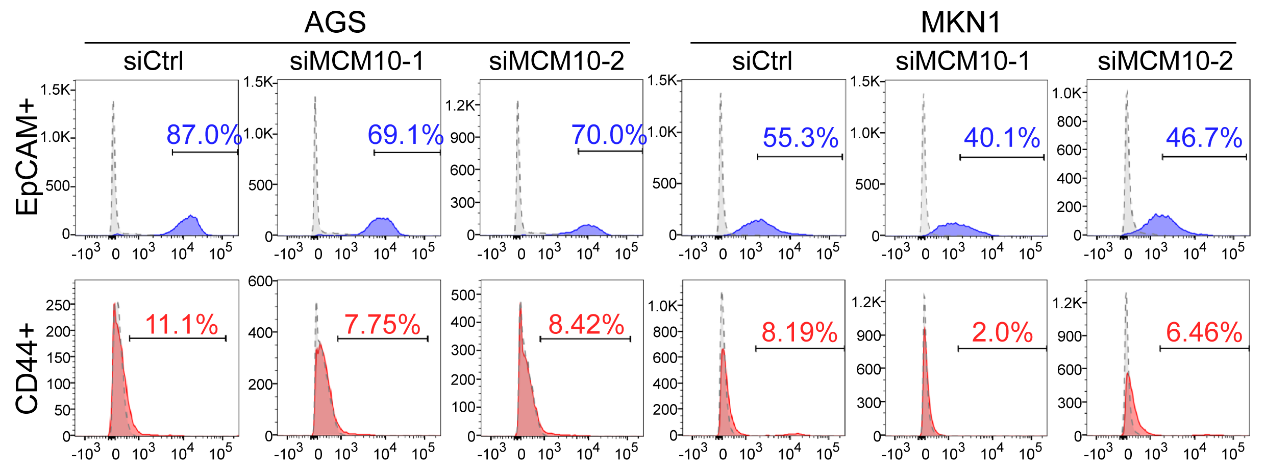
**

**Supplementary Figure S4** Flow cytometry assay revealing decreases in the percentages of cells expressing EpCAM and CD44 in MCM10-delepted cells.

**Supplementary Figure S5**


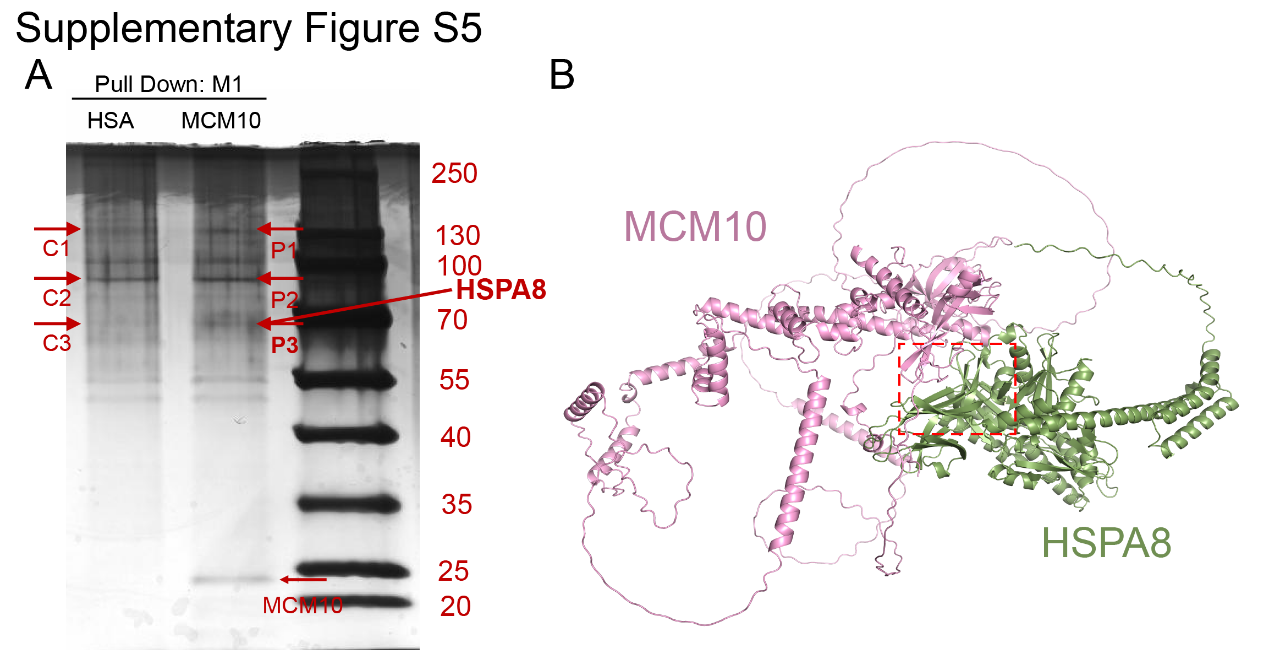


**Supplementary Figure S5** IP-MS analysis highlights HSPA8 as the potential mediator within the MCM10-β-catenin regulatory axis. **(A)** Human recombinant his-tagged MCM10 protein based pull-down assay and human serum albumin (HSA) was used as control. The obtained proteins were separated in 10% SDS-PAGE gel flowed by silver staining. **(B)** 3D modeling of the predicted MCM10-HSPA8 binding pattern and interacting interface.

**Supplementary Figure S6**

**
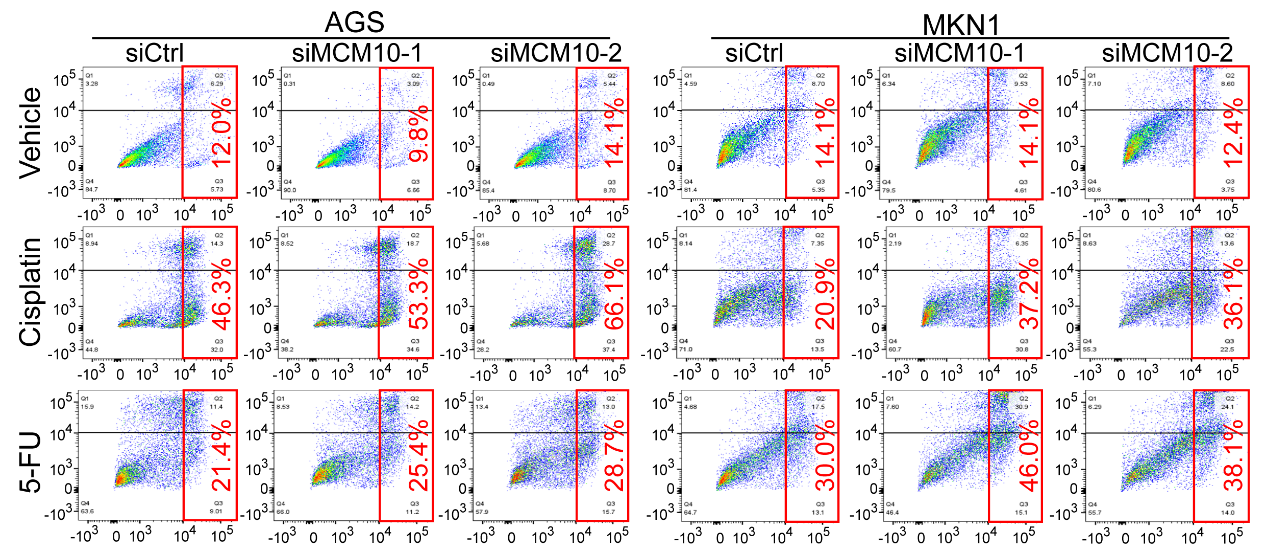
**

**Supplementary Figure S6** Annexin V assay demonstrating increased apoptosis of AGS and MKN1 cells after MCM10 depletion.

**Supplementary Figure S7**


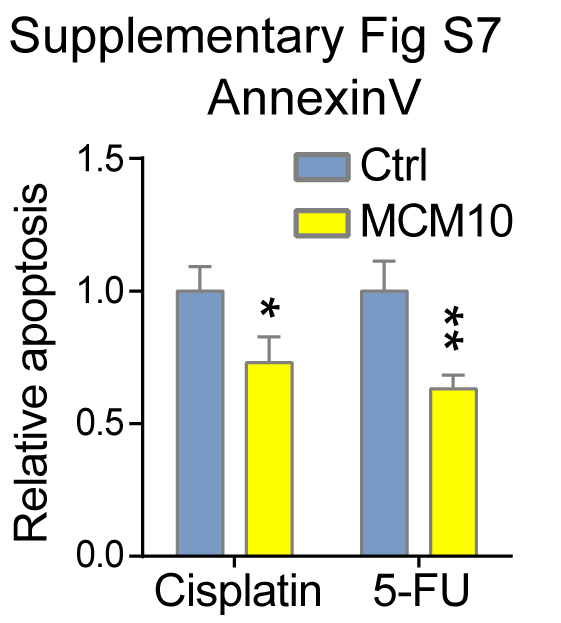


**Supplementary Figure S7** Annexin V assay demonstrates decreased apoptosis of MCM10-overexpressed MKN28 cells after either cisplatin or 5-FU treatment (n = 3).

**Supplementary Figure S8**

**
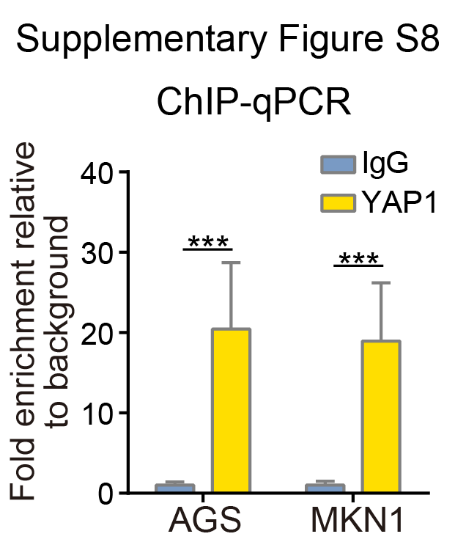
**

**Supplementary Figure S8** The ChIP-qPCR analysis confirmed significant enrichment of YAP1 at the same putative TEAD-binding site in the MCM10 promoter region (n = 3).

**Supplementary Figure S9**


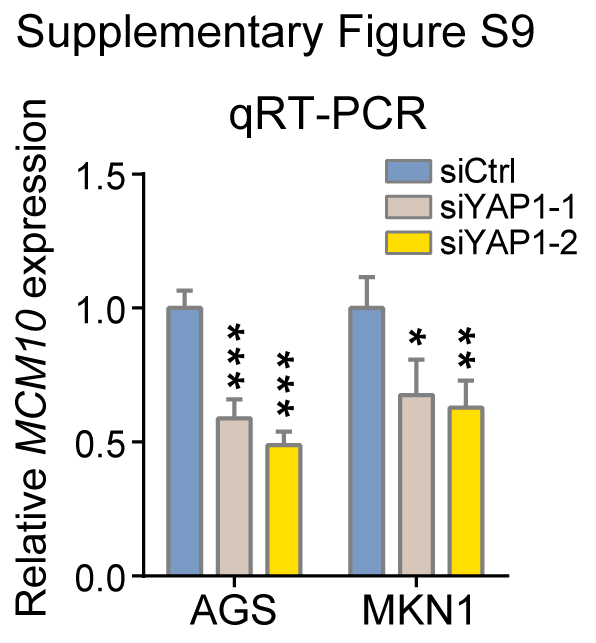


**Supplementary Figure S9** The depletion of YAP1 led to a significant downregulation of *MCM10* mRNA levels (n = 5).

**Supplementary Figure S10**

**
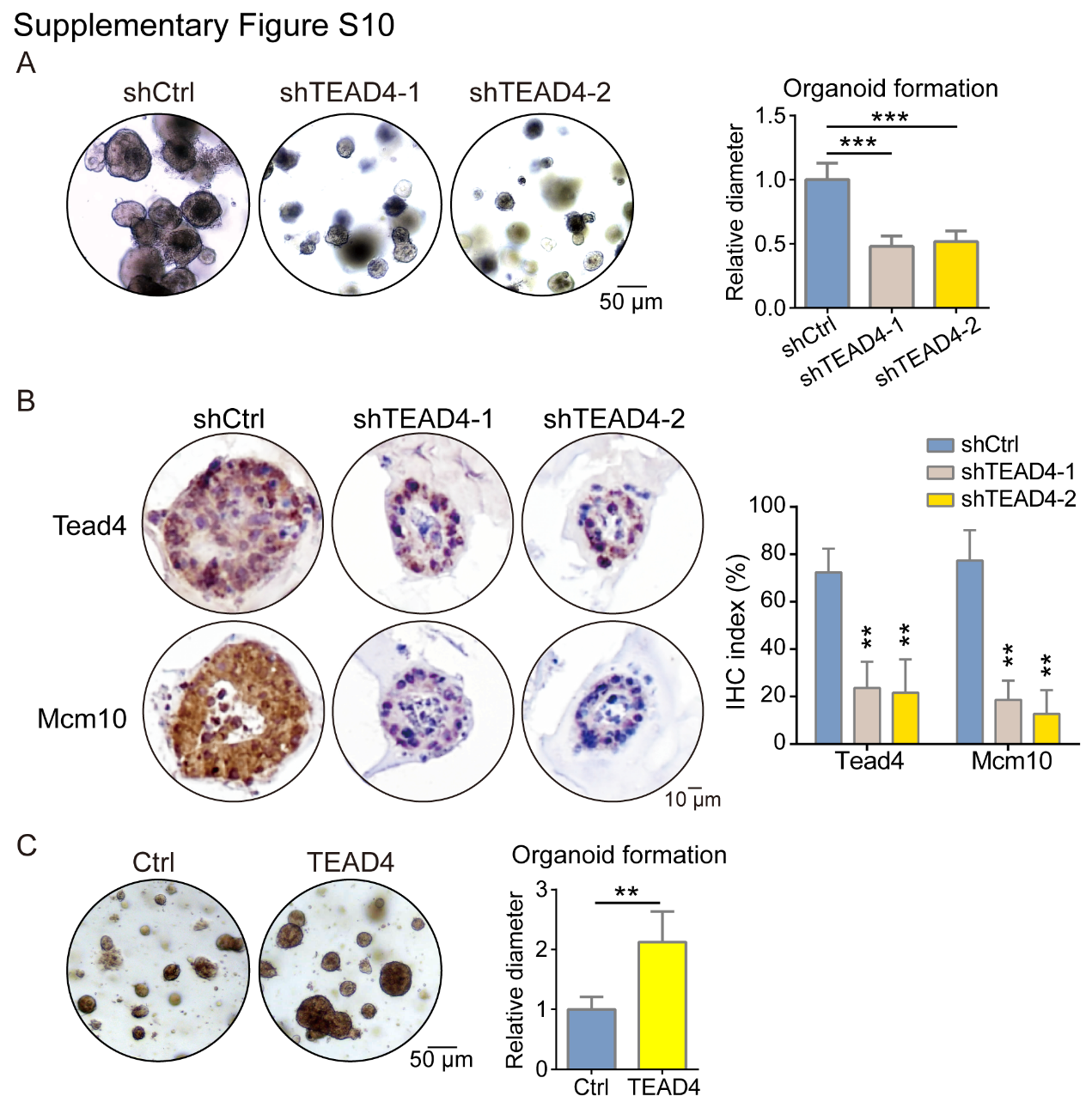
**

**Supplementary Figure S10** The oncogenic function of Tead4 and the regulation on Mcm10 are verified in mouse gastric organoid models. **(A)** Tead4 depletion impaired the growth ability of gastric organoids derived from TFF1-KO mice (n = 5). **(B)** Tead4 depletion downregulated Mcm10 protein expression in a murine context (n = 3). **(C)** Tead4 overexpression enhanced organoid proliferative capacity (n = 5).

**Supplementary Figure S11**

**
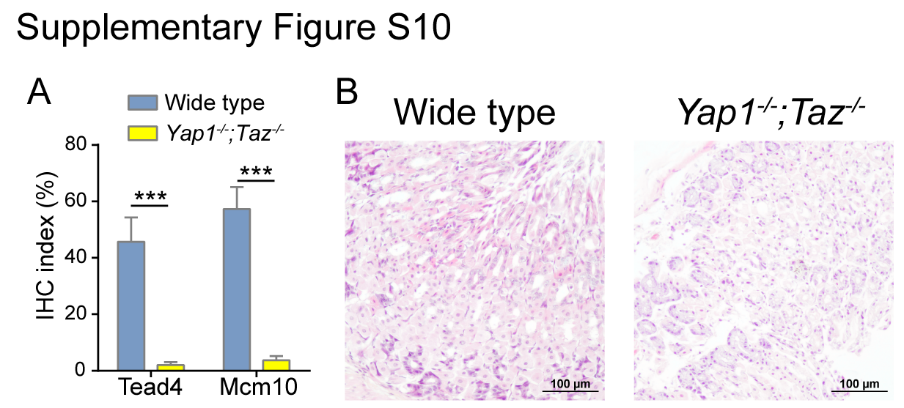
**

**Supplementary Figure S11** *Yap1^-/-^;Taz^-/-^* transgenic mice demonstrated significant decrease in Tead4 and Mcm10 levels. **(A)** Quantitative analyses on the IHC data from our transgenic mouse models. **(B)** H&E staining of gastric epithelial area from both control and tamoxifen-induced mice revealed no significant architectural disruption, inflammation, or metaplastic changes.

**Supplementary Figure S12**


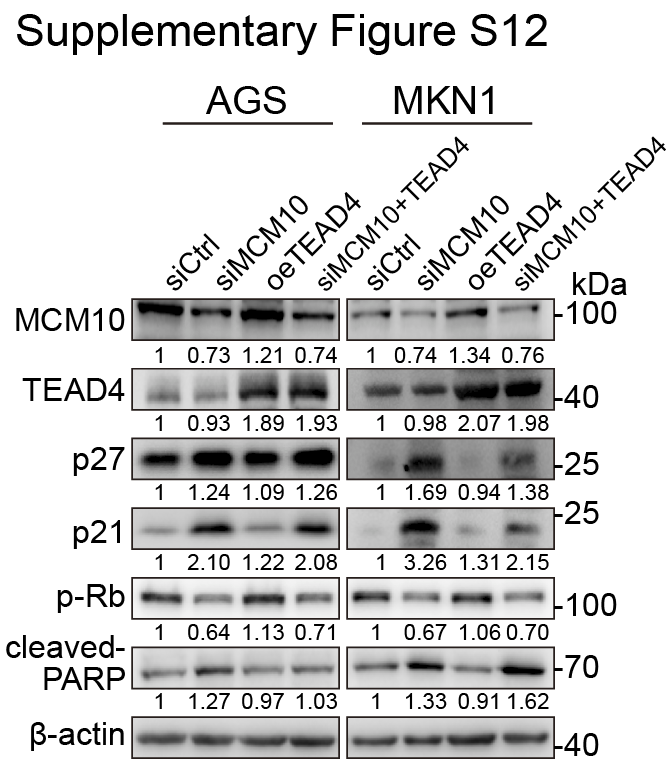


**Supplementary Figure S12** Western blot analysis on cell cycle-related markers upon MCM10-depletion and/or TEAD4 overexpression.

**Supplementary Figure S13**

**
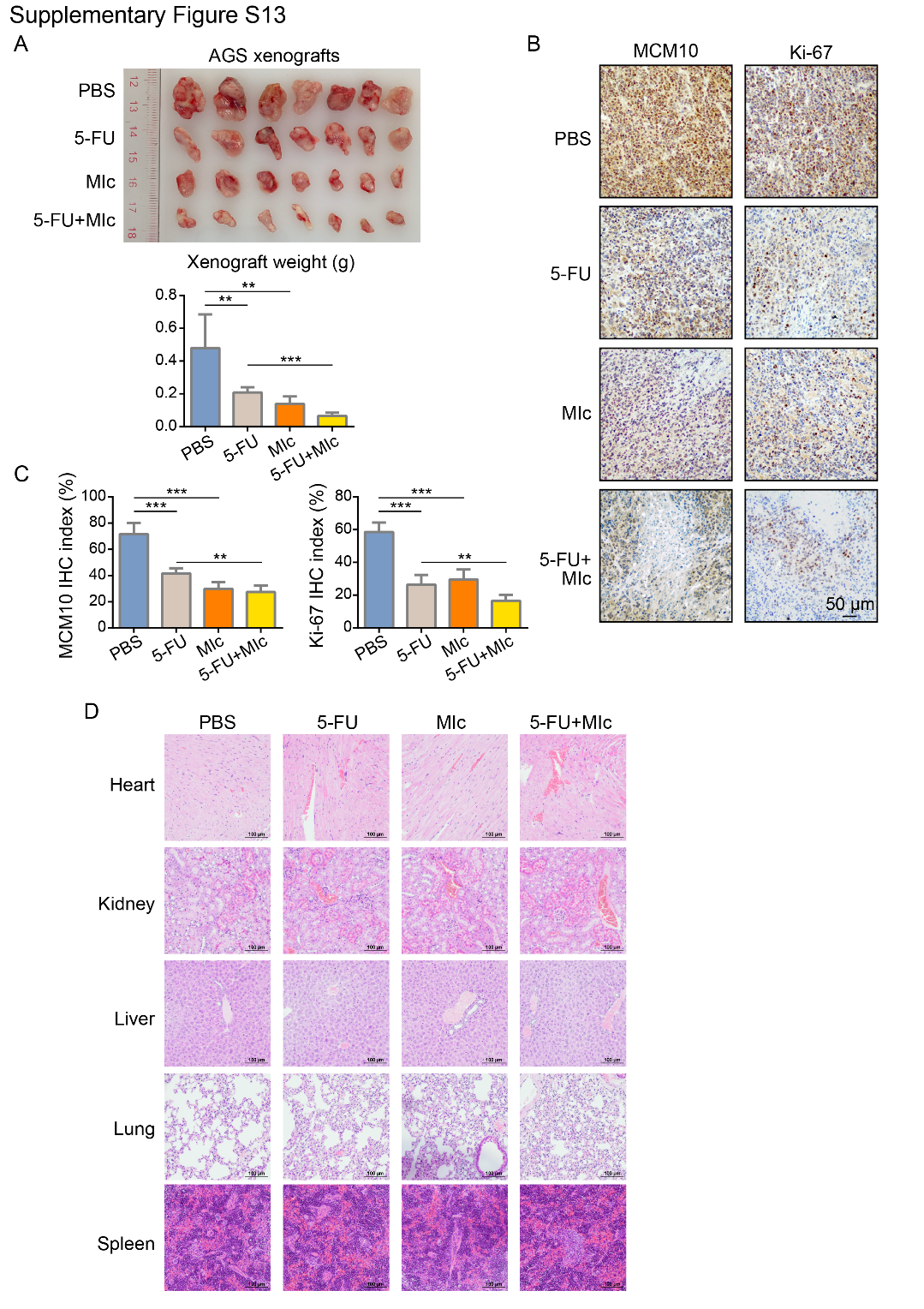
**

**Supplementary Figure S13** *In vivo* assessments of the efficacy, specificity, and safety profiles of MIc treatment. **(A)** Administration of MIc significantly impaired the growth of AGS-derived xenografts in NSG mice. MIc treatment markedly enhanced the anti-tumor efficacy of 5-FU. **(B)** Tumors from the MIc-treated group showed a significant reduction in nuclear MCM10 protein expression. **(C)** H&E staining of major organs (heart, liver, spleen, lung, and kidney) harvested from mice after the 28-day treatment period revealed no evidence of severe morphological damage or overt toxicity.

**References**

1. Xie F, Lyu Y, Chen B, Leung HW, Yu P, Feng T, Fang C, Cheung AHK, Zhou B, Jiang J, et al: **STK3 is a transcriptional target of YAP1 and a hub component in the crosstalk between Hippo and Wnt signaling pathways during gastric carcinogenesis.** *Mol Cancer* 2025, **24:**186.

2. van de Wetering M, Cavallo R, Dooijes D, van Beest M, van Es J, Loureiro J, Ypma A, Hursh D, Jones T, Bejsovec A, et al: **Armadillo coactivates transcription driven by the product of the Drosophila segment polarity gene dTCF.** *Cell* 1997, **88:**789-799.
